# Improved methane mitigation potential and modulated methane cycling microbial communities in arable soil by compost addition

**DOI:** 10.1093/ismeco/ycaf139

**Published:** 2025-05-29

**Authors:** Stijn G van den Bergh, Iris Chardon, Marion Meima-Franke, Germán Pérez, Gabriel S Rocha, Kristof Brenzinger, Gerard W Korthals, Jochen Mayer, Mathias Cougnon, Dirk Reheul, Wietse de Boer, Paul L E Bodelier

**Affiliations:** Department of Microbial Ecology, Netherlands Institute of Ecology (NIOO-KNAW), PO Box 50, 6700AB Wageningen, the Netherlands; Soil Biology Group, Wageningen University and Research, PO Box 47, 6700AA Wageningen, the Netherlands; Department of Microbial Ecology, Netherlands Institute of Ecology (NIOO-KNAW), PO Box 50, 6700AB Wageningen, the Netherlands; Department of Microbial Ecology, Netherlands Institute of Ecology (NIOO-KNAW), PO Box 50, 6700AB Wageningen, the Netherlands; Department of Microbial Ecology, Netherlands Institute of Ecology (NIOO-KNAW), PO Box 50, 6700AB Wageningen, the Netherlands; Laboratory of Microbiology, Department of Plant Biology, Agronomy Faculty, University of the Republic, Montevideo 12900, Uruguay; Department of Microbial Ecology, Netherlands Institute of Ecology (NIOO-KNAW), PO Box 50, 6700AB Wageningen, the Netherlands; Ecology and Biodiversity, Utrecht University, Padualaan 8, 3584 CH, Utrecht, the Netherlands; Department of Microbial Ecology, Netherlands Institute of Ecology (NIOO-KNAW), PO Box 50, 6700AB Wageningen, the Netherlands; Biointeractions and Plant Health, Wageningen Plant Research, PO Box 16, 6700AA Wageningen, the Netherlands; Agroecology and Environment, Agroscope, Reckenholzstrasse 191, 8046 Zürich, Switzerland; Plant Department, ILVO (Flanders Research Institute for Agriculture, Fisheries and Food), Caritasstraat 39, 9090 Melle, Belgium; Sustainable Crop Production, Department of Plants and Crops, Ghent University, 9000 Ghent, Belgium; Department of Microbial Ecology, Netherlands Institute of Ecology (NIOO-KNAW), PO Box 50, 6700AB Wageningen, the Netherlands; Soil Biology Group, Wageningen University and Research, PO Box 47, 6700AA Wageningen, the Netherlands; Department of Microbial Ecology, Netherlands Institute of Ecology (NIOO-KNAW), PO Box 50, 6700AB Wageningen, the Netherlands

**Keywords:** agricultural soil, compost, methane, methanotrophs, organic amendments, soil methane uptake potential

## Abstract

The global atmospheric concentration of the potent greenhouse gas methane (CH_4_) is rising rapidly, and agriculture is responsible for 30%–50% of the yearly CH_4_ emissions. To limit its global warming effects, strong and sustained reductions are needed. Sustainable agricultural management strategies, as the use of organic amendments like compost, have previously proven to have a potent CH_4_ mitigation effect in laboratory experiments. Here we investigated, using an extensive field study, the effect of organic amendments on the CH_4_ mitigation potential and CH_4_ cycling microbial communities of arable soils. Organic-amended soils had higher potential CH_4_ uptake rates and an improved potential to oxidize CH_4_ to sub-atmospheric concentrations. Also, we showed for the first time that the methanotrophic and methanogenic microbial communities of arable soils were unequivocally altered after organic amendment application by increasing in size while getting less diverse. Compost-amended soils became dominated by the compost-originating methanotroph *Methylocaldum szegediense* and methanogen *Methanosarcina horonobensis*, replacing the indigenous methane cycling community members. However, multivariate analyses didn’t point out type Ib methanotrophs like *M. szegediense* as significant driving factors for the observed improved soil CH_4_ uptake potential. Conventional type IIa methanotrophs like *Methylocystis* sp. also had higher differential abundances in organic-amended soils and are speculated to contribute to the improved CH_4_ uptake potential. Altogether, the results showed that compost serves as a vector for the introduction of CH_4_ cycling microbes and improves the soil’s CH_4_ uptake potential, which emphasizes the potential of organic fertilization with compost to contribute to CH_4_ mitigation in agricultural soils.

## Introduction

Methane (CH_4_) is a potent greenhouse gas (GHG) in Earth’s atmosphere that contributes substantially to global warming, as it has 34 times higher global warming potential (GWP) than carbon dioxide (CO_2_) on a 100-year time scale [1–3]. In fact, the current atmospheric concentration of 1.896 parts per million (ppm*_v_*) CH_4_ is the highest in at least 800 000 years and has increased by 156% since the industrial revolution [2]. This increase has been accelerating in recent years at an alarming rate [4]. Strong, rapid and sustained CH_4_ mitigation strategies are thus needed to limit global warming [1, 2]. Considering the global CH_4_ budget, an estimated 30%–50% of yearly CH_4_ emissions originate from agriculture, which includes emissions from both upland and rice paddy soils. Aerobic upland soils, however, are the only known biological sink for atmospheric CH_4_ [3, 5]. Yet, the CH_4_ uptake by agricultural soils is reduced 3–9 times compared to undisturbed upland soils because of conventional agricultural practices like ploughing, as that destroys the physical structure of the soil which is important for microbial consumption of atmospheric methane, and the use of nitrogen-rich mineral fertilizers [6–8].

The net CH_4_ flux of soils is a balance between CH_4_ production (methanogenesis) and oxidation (methanotrophy) [9]. During methanogenesis, CH_4_ is produced under anaerobic conditions by methanogenic Archaea (methanogens) as the product of organic matter decomposition or the reduction of CO_2_, acetate, and other C_1_-compounds. The phylogeny of methanogens, but also their environmental abundance, is largely based on the marker gene *mcrA*, encoding for the α subunit of the methyl-coenzyme M reductase, which is present in all methanogens and catalyzes the last step of the methanogenic metabolic pathway [10]. In upland soils, CH_4_ is generally oxidized by aerobic methane oxidizing bacteria (MOB), also known as methanotrophs [11, 12]. MOB are present in the phyla *Proteobacteria*, *Verrucomicrobia*, and *Actinobacteria*, and are characterized for having a methane monooxygenase (MMO) enzyme, either in particulate or soluble form, whose major subunits are encoded for by *pmoA* or *mmoX*, respectively [13–15]. Historically, proteobacterial MOB have been divided into two major groups, type I and type II methanotrophs, respectively affiliated with the classes *Gammaproteobacteria* and *Alphaproteobacteria*, based on distinctive features like the carbon fixation mechanism [12, 16]. Today, MOB are still classified in designated types, based on *pmoA* as phylogenetic marker [13]. Aerobic MOB convert CH_4_ into methanol (CH_3_OH) using oxygen as primary electron acceptor, as the first step of the CH_4_ oxidation metabolic pathway, eventually converting the carbon either assimilatory into biomass or dissimilatory into CO_2_ [17]. Most MOB have a low-affinity for the oxidation of CH_4_, as they can only grow when oxidizing high concentrations of CH_4_ (>600 ppm*_v_*), and are so-called low-affinity methanotrophs. MOB with the intrinsic capability to grow when oxidizing CH_4_ at circum- and sub-atmospheric concentrations are likewise called high-affinity methanotrophs [18, 19]. High-affinity methanotrophs are mostly clustered in specific clades like Upland Soil Cluster α (USCα) and USCγ among others [14, 20], and did not have cultured representatives until recent years [21–23]. It has long been assumed that only high-affinity methanotrophs were responsible for the oxidation of CH_4_ at atmospheric concentrations, but also conventional, low-affinity MOB are capable of atmospheric CH_4_ oxidation after exposure to high concentrations of CH_4_ [19, 22, 24]. These conventional MOB store the energy derived from the oxidation of the high CH_4_ concentration spikes in storage compounds like polyhydroxybutyrate (PHB), which can be subsequently used to oxidize CH_4_ at low concentrations [19, 25–27]. However, recent work has demonstrated that cultured representatives among which *Methylocapsa* and *Methylocystis* species, have the capability of high affinity methane oxidation through physiological adaptation via gearing down C-assimilation, diverting all available energy to CH_4_ oxidation machinery as well as to the consumption of hydrogen and carbon monoxide [28].

Climate-smart sustainable agricultural strategies, like organic fertilization or non-inversion tillage, aim to minimize GHG emissions by enhancing carbon sequestration while maintaining or even enhancing soil fertility and productivity [29, 30]. Organic fertilization serves as an organic carbon and nitrogen input measure, improves the soil’s water retention capacity, water infiltration, and moisture content, and impacts the pH of the soil [31], all key factors affecting soil CH_4_ oxidation [32, 33]. Soil-based experiments under controlled conditions with prepared mixtures have shown that the use of organic amendments, such as compost or cover crop residue incorporation, can significantly enhance the CH_4_ uptake potential of agricultural soils, with compost being the most efficient amendment in terms of GWP [6, 24, 34–36]. The occurrence under field conditions, underlying mechanisms, and responsible MOB for this stimulation of atmospheric CH_4_ uptake by organic residues in agricultural soils remain to be elucidated. We hypothesize that organic amendment application increases the availability of trace metals and essential substrates like intracellular reducing equivalents required for CH_4_ oxidation in soils [31, 37, 38], thereby enhancing the soil CH_4_ uptake potential. Secondly, we hypothesize that via compost application MOB are introduced into the soil, which were potentially highly activated during the composting process [27]. It has been shown before that other relevant methane cycling microbes like methanogens can be introduced in soil via compost and manure [39, 40], and that microbial inoculants have a positive effect on the structure of soil microbial communities [41]. Therefore, in this study, the effects of different climate-smart management strategies (organic fertilization and non-inversion tillage) on the CH_4_ uptake potential and methane cycling microbial communities of arable soils were assessed. Furthermore, the controlling factors of both the CH_4_ uptake potential and microbial communities were determined, offering valuable insights on the CH_4_ mitigation potential of agricultural soils and climate-smart agricultural management strategies.

## Material and methods

### Field site description, soil sampling, and storage

To assess the effect of organic fertilization on the CH_4_ mitigation potential and methane cycling microbial communities of agricultural soils, a screening study was performed across existing field experiments in the Netherlands, Belgium, and Switzerland. To this end, six existing long-term field experiments (>5 years) with an organic fertilization treatment in a randomized block-design were included for this study, as described previously [42]. At each site, all available plots per organic treatment, i.e. a no organic fertilization control versus an organic-fertilized treatment (for five locations compost-amended and for one location cover crops-incorporated), were sampled (*n* = 4–6) [42]. At five sites, all plots received no structural additional inorganic fertilizer, and at location Melle all plots received 200 kg ha^−1^ y^−1^ mineral N fertilization. These sites represent common soil types and agricultural practices (regular tillage and non-inversion tillage (<5 cm), hereafter referred to as no-tillage) for northwestern Europe (Table 1). Soil samples (0–15 cm depth) were collected using stainless steel soil cores. To allow for the additional analysis of the upper-top and sub-top layers (0–7.5 cm and 7.5–15 cm depth, respectively), the samples were first subdivided into these layers, and subsequently mixed 1:1 to represent the whole 0–15 cm soil layer. All samples were sieved <2 mm, and sub-samples were stored at −20°C or oven dried at 40°C for later molecular and chemical analysis, respectively.

**Table 1:** Field site description and sampling information.

**Location**	**Coordinates**	**Sampling date**	**Agricultural practice**	**Organic treatment**	**Compost type**	**Application amount**	**Time since application**	**Application yearly since**	**Soil texture**	**Plot cover**	**Additional comments**	**References**
Vredepeel, Netherlands	51.542934, 5.849262	16-9-2019	No-tillage and tillage	No organic amendment and compost amendment	Green compost	10 ton ha^−1^ yr^−1^	177 days	2011	Sandy soil (sand 93.3%, silt 4.5%, clay 2.2%)	Leek	N2K20 fertilization 10 days prior.	[43]
Valthermond, Netherlands	52.877470, 6.929775	15-4-2019	No-tillage	No organic amendment and compost amendment	Green compost	20 ton ha^−1^ yr^−1^	12 days	2013	Sandy peat soil (sand 90%, silt 7%, clay 3%)	Bare soil	Reclaimed peat, now sandy soil.	[44]
Lelystad, Netherlands	52.545608, 5.578861	30-4-2019	No-tillage	No organic amendment and compost amendment	Green compost	20 ton ha^−1^ yr^−1^	41 days	2012	Clay loam soil (sand 66%, silt 12%, clay 23%)	Weeds	Weed control using Roundup Ultimate (3 L ha^−1^) 30 days prior.	[45]
Melle, Belgium	50.979721, 3.816310	14-5-2019	Tillage	No organic amendment and compost amendment	Green compost	50 m^3^ ha^−1^ yr^−1^ (≈ 20 ton ha^−1^ yr^−1^)	7 days	2004	Loamy sand soil (sand 79.8%, silt 11.6%, clay 8.6%)	Fodder beet or forage maize	Yearly 200 kg ha^−1^ mineral nitrogen fertilization.	[46]
Wageningen, Netherlands	51.995069, 5.659699	16-3-2020	Tillage	No organic amendment and cover crops incorporation	*n/a*	approx. 5 ton ha^−1^ yr ^−1^	14 days	2016	Sandy soil (sand 83%, silt 12%, clay 2%)	Bare soil	Cover crop mixture (1:1:1) of *Raphanus sativus* (radish), *Avena strigosa* (oat), and *Vicia sativa* (vetch).	[47]
Zürich, Switzerland	47.428737, 8.516487	12-10-2021	Tillage	No organic amendment and compost amendment	Green compost	2.5 ton dry org. Matter ha^−1^ yr^−1^ (approx. 8 ton ha^−1^ yr^−1^)	8 days	1949	Sandy loam soil (sand 57%, silt 27%, clay 14%)	Bare soil	Weed and pest control using herbicides and fungicides during whole growing season (last 55 days prior).	[48]

### Physicochemical properties

The moisture content was measured on basis of loss of weight after oven-drying ~10 g of soil at 105°C for 24 h, after which the organic matter content was measured as loss of weight after burning the dried samples in an oven at 430°C for 24 h [49]. The maximum water holding capacity was determined using the funnel method [50]. The pH was measured in water suspension (1:2.5, *w/v*) after shaking for 2 h. The NH_4_^+^-N (ammonium) and the sum of NO_2_^-^-N (nitrite) and NO_3_^-^-N (nitrate) (hereafter (NO_2_^−^ + NO_3_^−^)-N content) was determined in a 1 M KCl-extract (1:5, *w/v*) [51] using a SEAL QuAAtro Segmented Flow Analyzer (Beun-de Ronde B.V., Abcoude, the Netherlands), and a selection of the bioavailable elemental contents were determined in an acidified 0.01 M CaCl_2_-extract (1:10, *w/v;* 1% *v/v* HNO_3_) [52] using an ICP-OES 6500 DUO (Thermo Fisher Scientific, Bleiswijk, the Netherlands), as described previously [27]. Also, the particle size distribution (PSD) using laser diffraction analysis (LDA) was determined, of which a detailed description is provided in the Supplemental materials & methods.

### Potential methane oxidation rates

The potential methane uptake rates at near-atmospheric and high CH_4_ concentrations were determined by incubating 10 g of fresh soil in a 60 mL glass serum bottle with an initial headspace CH_4_ concentration of ~10 or ~ 10 000 ppm*_v_*, respectively. CH_4_ headspace concentration was measured by injecting headspace samples in an Ultra gas chromatograph (GC; Interscience, Breda, The Netherlands) equipped with a Flame Ionization Detector (FID) and a Rt-Q-Bond (L 30 m, ID 0.32 mm, df 10 μm; Restek, Interscience) capillary column, as described previously [27]. One-point calibrations were performed with certified gas mixtures of 10 ppm*_v_* and 990 ppm*_v_* CH_4_ in N_2_ (Westfalen, Deventer, The Netherlands). Chromeleon™ Chromatography Data System 7.1 software (Thermo Fisher Scientific) was used to process the obtained chromatograms from the GC. The methane uptake rates were determined by linear regression of the CH_4_ depletion curves for both CH_4_ concentrations (*p ≤* .05). The lag phase was calculated as the intersection of the linear regressions of the CH_4_ oxidation and the preceding inactive phase.

### DNA extraction and qPCR assays

DNA was extracted from approximately 0.5 g of soil, freeze dried using an Alpha 2-4 LD freeze dryer (Martin Christ, Osterode am Harz, Germany), using the DNeasy PowerSoil Pro Kit (Qiagen, Venlo, The Netherlands) according to manufacturer’s instruction. A NanoDrop One Spectrophotometer (Thermo Fisher Scientific) was used to measure the quality and quantity of the extracted DNA, which was stored at −20°C until further molecular analyses. Quantitative PCR (qPCR) assays were performed targeting the 16S rRNA gene for Archaea and Bacteria, the *pmoA* gene for methanotrophs, and the *mcrA* gene for methanogens as described previously [27], using a CFX96 Touch Real-Time PCR System (Bio-Rad Laboratories, Lunteren, The Netherlands). The PCR reaction compositions and profiles for all primer pairs and amplification efficiencies are further described in the Supplemental materials & methods and Table S1, S2.

### 16S rRNA, *pmoA* and *mcrA* gene amplicon sequencing and analysis

Amplicon sequencing was performed on all DNA extracts, targeting the bacterial 16S rRNA gene, *pmoA* for methanotrophs with a nested multiplex-reverse approach, and *mcrA* for methanogens (Table S3). Further processing of the extracted DNA, library construction, amplification and Illumina MiSeq or NextSeq PE250 (16S rRNA and *mcrA*) or PE300 (*pmoA*) sequencing was done by the Genome Québec Centre d’expertise et de services (Quebec, Canada). Raw sequences can be found at the European Nucleotide Archive under the accession number PRJEB75729 (http://www.ebi.ac.uk/ena/data/view/PRJEB75729).

Processing of raw sequencing data was done using the DADA2-plugin in QIIME2 (version 2020.2) [53, 54] as described previously [27]. Classification was done based on the SILVA nonredundant Small Subunit rRNA database (version 138.1) for 16S [55], and on custom-built reference databases based on NCBI sequences [56] for *pmoA* and *mcrA*, of which the prior was complemented with sequences from Knief (2015) [13, 27, 57]. All downstream analyses were performed in R (version 4.3.2) [58] using the *phyloseq* and *microeco* packages [59, 60]. The number of reads in each sample was normalized using the median sequencing depth. A Random Forest analysis combined with a Wilcoxon rank sum test was used to reveal differentially abundant species, and the alpha and beta diversity were assessed on basis of the Shannon index and PCoA ordination using Bray–Curtis distances, respectively [60].

### Statistical analyses

All statistical analyses were done using R (version 4.3.2) [58]. Multivariate redundancy analyses (RDAs) were performed to determine controlling factors of methane uptake rates and to analyze methanotrophic and methanogenic community composition-environment relationships, using the *vegan* package in R [61]. A Pearson correlation analysis between the relative and differential abundances of methanotrophic species and environmental variables was performed using the *microeco* package [60]. A detailed description of the statistical analyses is provided in the Supplemental materials & methods.

## Results

### Physicochemical properties

The soil physicochemical properties are shown in Table S4. The full descriptive statistics are shown in Table S5 for the organic treatment effect (organic-amended vs unamended), location effect, agricultural practice effect (tillage vs no-tillage) and the interaction between the latter two and the organic treatment, as well as a pairwise comparison between the soil and its sublayers. The pH was higher in organic-amended soils than in unamended soils (Scheirer–Ray–Hare test, *p* ≤ .05). The moisture content was higher in no-tilled soils and their upper-top layer (ANOVA and Scheirer-Ray-Hare test, respectively, both *p* ≤ .05), while there were no treatment or practice effects in the sub-top layer. At two locations (Melle and Zürich), the organic-amended soils had a higher organic matter content than the unamended soils, as well as in both sublayers, but there was no overall organic treatment effect. A further analysis of the physicochemical properties of the upper-top and sub-top layers and of individual locations, and a detailed analysis and discussion of the PSD using LDA (Table S6), is provided in the Supplemental results.

### Methane uptake potential of agricultural soils

**Figure 1:**
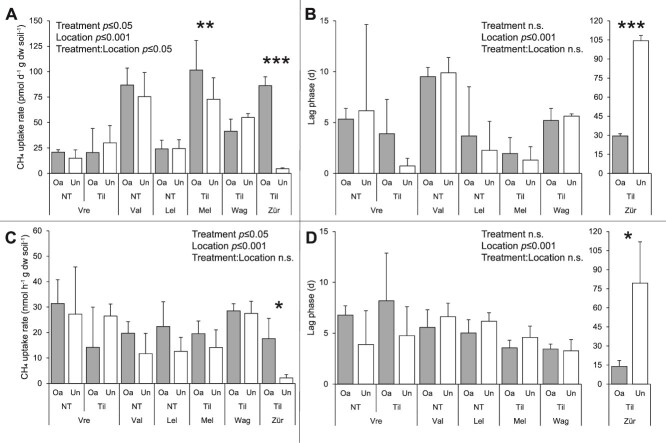
Potential CH_4_ uptake rates (A, C) and lag phases (B, D) in incubations with ~10 ppm_*v*_ (A, B) and ~10 000 ppm_*v*_ (C, D) CH_4_ (mean ± SD; *n* = 4–6) of agricultural soils (0–15 cm depth) of visited field sites (Vre—Vredepeel; Val—Valthermond; Lel—Lelystad; Mel—Melle; Wag—Wageningen; Zür—Zürich), varying in organic treatment (Oa—Organic-amended; Un—Unamended) and agricultural practice (NT—No-tillage; Til—Tillage). Significant differences within a location are indicated with an asterisk (paired t-test; ^*^p ≤ .05; ^**^p ≤ .01; ^***^p ≤ .001). Treatment and location effects, and their interaction, are given if significant (one-way ANOVA or Scheirer–Ray–Hare test).

In the near-atmospheric CH_4_ incubation (~10 ppm*_v_*), organic-amended soils had a significantly higher potential methane uptake rate than unamended soils (Scheirer–Ray–Hare test, *p* ≤ .05; Fig. 1A). This was mainly driven by significantly enhanced rates at locations Melle and Zürich, also reflected in a significant interaction between treatment and location (Scheirer–Ray–Hare test, *p* ≤ .05; Table S5). While the organic-amended upper-top layer had a higher potential methane uptake rate (Scheirer–Ray–Hare test, *p* ≤ .05; Fig. S1a), the sub-top layer did not (Fig. S2A), and for both soil layers there was a significant interaction between treatment and location (Scheirer–Ray–Hare test, both *p* ≤ .01; Table S5). Furthermore, there was no overall effect of the organic treatment or tillage practice on the lag phase, but organic-amended Zürich soil had a notable shorter lag phase than its unamended counterpart (Fig. 1B). Also, there were no overall effects on the potential to oxidize to sub-atmospheric CH_4_ concentrations (<1.89 ppm*_v_*) (Table S5, S7).

In the high CH_4_ incubation (~10 000 ppm*_v_*), organic-amended soil had a significantly higher potential methane uptake rate than unamended soils (ANOVA, *p* ≤ .05; Fig. 1C), and there was no significant interaction between treatment and location (Table S5). Furthermore, there was no treatment effect on the lag phase of the soil, despite a significantly reduced lag phase at Zürich (Fig. 1D). For the upper-top layer there were no overall effects (Figure S1C, D), but the organic-amended sub-top layer had a higher potential methane uptake rate (Scheirer–Ray–Hare test, *p* ≤ .05; Fig. S2C), while there was no significant interaction between treatment and location (Table S5). Additionally, the organic-amended soil showed a significantly higher potential to oxidize CH_4_ to <10 ppm*_v_* and sub-atmospheric CH_4_ concentrations (χ^2^-test, *p* ≤ .01 and *p* ≤ .05, respectively; Table S5, S7), again without a significant interaction between treatment and location. In both incubations with near-atmospheric and high CH_4_ concentrations, the potential methane uptake rates were mainly driven by the activity in the sub-top layer. The upper-top layer, however, had a better potential to oxidize CH_4_ to <10 ppm*_v_* and sub-atmospheric concentrations in both incubations (Table S5, S7). A more detailed analysis of the potential methane uptake rates of the upper-top and sub-top layers and individual locations is provided in the Supplemental results.

### Abundance analyses of methane cycling bacteria and archaea

**Figure 2:**
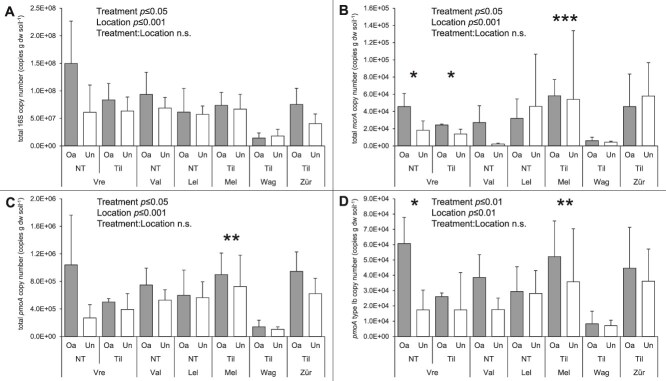
Abundance of 16S (A), *mcrA* (B), total *pmoA* (C), and *pmoA* type Ib (D) genes per gram dry weight soil (mean ± SD; *n* = 4–6) of agricultural soils (0–15 cm depth) of visited field sites (Vre—Vredepeel; Val—Valthermond; Lel—Lelystad; Mel—Melle; Wag—Wageningen; Zür—Zürich), varying in organic treatment (Oa—Organic-amended; Un—Unamended) and agricultural practice (NT—No-tillage; Til—Tillage). Significant differences within a location are indicated with an asterisk (paired t-test; ^*^p ≤ .05; ^**^p ≤ .01; ^***^p ≤ .001). Treatment and location effects, and their interaction, are given if significant (one-way ANOVA or Scheirer-Ray-Hare test).

Organic-amended soils had a higher number of bacterial 16S rRNA, *mcrA*, and *pmoA* gene copies than unamended soils (Scheirer–Ray–Hare test, all *p* ≤ .05), and for all genes there was no significant interaction between treatment and location (Table S5). More specifically, organic-amended soils had a higher number of type Ib *pmoA* gene copies, up to 5.74×10^4^ copies g dw soil^−1^ (Scheirer–Ray–Hare test, *p* ≤ .01; Fig. 2), while concurrently, there was no difference between organic- and unamended soils for type II *pmoA* gene copy numbers, on average 1.06×10^7^ and 8.96×10^6^ copies g dw soil^−1^, respectively (Fig. S3). For both type Ib and type II *pmoA* gene copy numbers there was no significant interaction between treatment and location (Table S5). Considering individual locations, only organic-amended Melle soil had higher *pmoA* (total and type Ib) and *mcrA* copy numbers than its unamended counterpart. Furthermore, there was no effect of tillage practice on the observed copy numbers of all genes (Table S5).

In both the upper-top layer (Fig. S4) and the sub-top layer (Fig. S5), the type Ib *pmoA* copy numbers were significantly higher in organic-amended soils than in unamended soils (ANOVA, *p* ≤ .01 and *p* ≤ .001, respectively), without a significant interaction between treatment and location. In the sub-top layer, the total *pmoA* gene copy numbers were also significantly higher in the organic-amended soils than in the unamended soils (ANOVA, *p* ≤ .05), again without a significant interaction between treatment and location. Furthermore, the upper-top layer had a higher bacterial 16S rRNA and *pmoA* type Ib copy number than the sub-top layer (Dunn’s test and Tukey’s HSD test, respectively, both *p* ≤ .05; Table S5). A more detailed analysis of the gene abundances of relevant microbial groups of the upper-top and sub-top layers and individual locations is provided in the Supplemental results.

### Total bacterial, methanotrophic, and methanogenic community composition analyses

The composition of the relevant bacterial and archaeal communities was determined using amplicon sequencing analysis of the bacterial 16S rRNA, *pmoA* and *mcrA* genes, and the methanotrophic and methanogenic communities of organic-amended soils became less diverse and mostly dominated by single species. After normalization, 32 882, 5827 and 5075 amplicon sequencing variants (ASV) were identified and classified of the bacterial 16S rRNA, *pmoA* and *mcrA* genes, respectively (Fig. S6). The total bacterial communities of the soil (Fig. S7), the upper-top layer (Fig. S8), and the sub-top layer (Fig. S9) were dominated by six phyla together making up well over 75% of the total bacterial community in all soils, of which the phylum *Proteobacteria* had a relative abundance of 10%–20%. The phylum *Methylomirabilota* had a relative abundance between 0.1 and 3.0% in all soils (Fig. S7A). Overall, both the alpha- and beta diversity of the soil bacterial community was not affected by the organic treatment (Figure S7B, C, Table S8).

**Figure 3:**
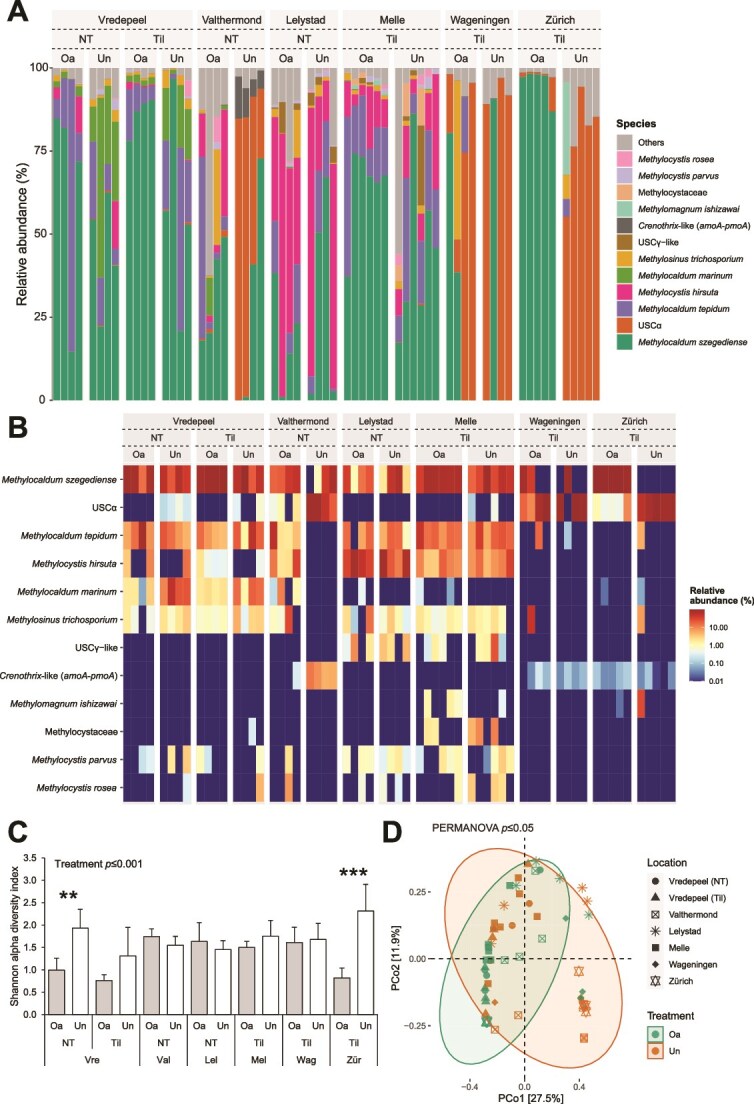
Relative abundance of the methanotrophic community at the species level (A), heatmap of most abundant methanotrophic species (B), Shannon alpha diversity (C), and PCoA beta diversity (D) based on *pmoA* ASV’s of agricultural soils (0–15 cm depth) of visited field sites (Vre—Vredepeel; Val—Valthermond; Lel—Lelystad; Mel—Melle; Wag—Wageningen; Zür—Zürich), varying in organic treatment (Oa—Organic-amended; Un—Unamended) and agricultural practice (NT—No-tillage; Til—Tillage). Significant differences on the alpha diversity within a location are indicated with an asterisk (paired t-test; ^*^p ≤ .05; ^**^p ≤ .01; ^***^p ≤ .001), and overall treatment effects on the alpha and beta diversity are given (one-way ANOVA and PERMANOVA, respectively).

The methanotrophic communities of the soil (Fig. 3), the upper-top layer (Fig. S10), and the sub-top layer (Fig. S11) were dominated by *Methylocaldum* species (mainly *Methylocaldum szegediense*), USCα, and *Methylocystis hirsuta*. *M. szegediense* was dominantly abundant in most of the soils, and its relative abundance was higher in soils with organic amendment application (ANOVA, *p* ≤ .01). Conversely, at three locations, USCα was the dominant methanotrophic species in unamended soils (ANOVA, *p* ≤ .01; Fig. 3A and B). Moreover, *M. szegediense* and *Methylosinus trichosporium* had a significantly higher differential abundance in organic-amended soils (Random Forest, *p* ≤ .01 and *p* ≤ .05, respectively), while USCα had a higher differential abundance in unamended soils (Random Forest, *p* ≤ .05; Table S9). Furthermore, the overall alpha diversity of the methanotrophic community was lower in organic-amended soil than in unamended soil (ANOVA, *p* ≤ .001), and the beta diversity was significantly different between organic- and unamended soils (PERMANOVA, *p* ≤ .05, R^2^ = .03; Fig. 3C, D and Table S8).

**Figure 4:**
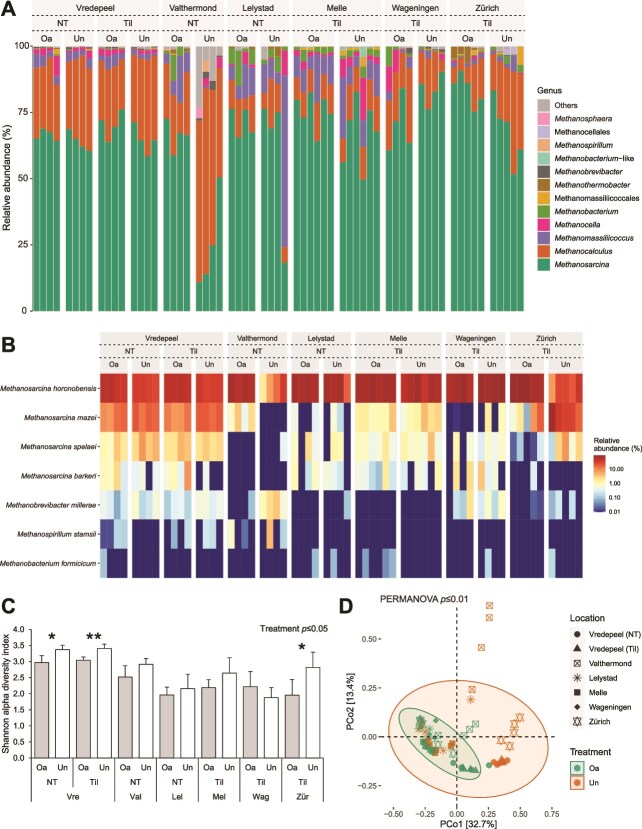
Relative abundance of the methanogenic community at the genus level (A), heatmap of most abundant methanogenic species (B), Shannon alpha diversity (C), and PCoA beta diversity (D) based on *mcrA* ASV’s of agricultural soils (0–15 cm depth) of visited field sites (Vre—Vredepeel; Val—Valthermond; Lel—Lelystad; Mel—Melle; Wag—Wageningen; Zür—Zürich), varying in organic treatment (Oa—Organic-amended; Un—Unamended) and agricultural practice (NT—No-tillage; Til—Tillage). Significant differences on the alpha diversity within a location are indicated with an asterisk (paired t-test; ^*^p ≤ .05; ^**^p ≤ .01; ^***^p ≤ .001), and overall treatment effects on the alpha and beta diversity are given (one-way ANOVA and PERMANOVA, respectively).

The methanogenic communities of the soil (Fig. 4), the upper-top layer (Fig. S12), and the sub-top layer (Fig. S13) were dominated by the genus *Methanosarcina*, and to a lesser extent by *Methanocalculus* and *Methanomassiliicoccus*, together making up >75% of the methanogenic community at all locations. More specifically, *Methanosarcina horonobensis* was the most dominant methanogenic species, having a higher relative and differential abundance in organic-amended soils (ANOVA, *p* ≤ .001; Random Forest, *p* ≤ .001; Table S9). Overall, in organic-amended soils the alpha diversity of the methanogenic community was lower than in unamended soils (ANOVA, *p* ≤ .05), and the beta diversity of organic- and unamended soils was significantly different (PERMANOVA, *p* ≤ .01, R^2^ = .07; Fig. 4C, D and Table S8).

The effect of the organic amendment application on the methanotrophic and methanogenic communities was more apparent in the upper-top layer than in the sub-top layer of the soil, as both the alpha and beta diversity were significantly lower and different, respectively, within more individual locations, next to significant overall effects. A more detailed analysis of the total bacterial, methanotrophic and methanogenic communities of the upper-top and sub-top layers and individual locations is provided in the Supplemental results.

### Potential controlling factors of the methane uptake potential and associated microbial communities

**Figure 5:**
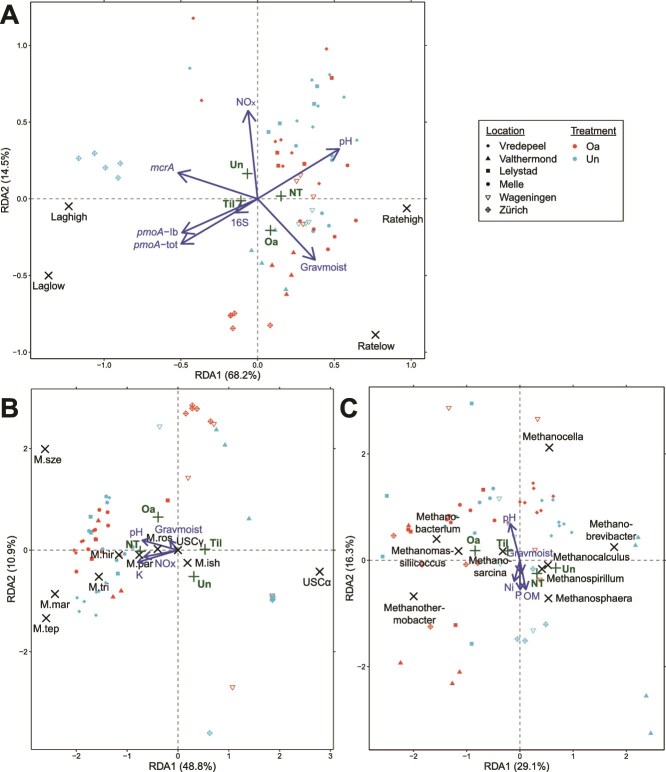
RDA on variables explaining the variability observed in the methane uptake potential (A), the methanotrophic community (B) and the methanogenic community (C) in agricultural soils (0–15 cm depth), obtained using the environmental variables gravimetric moisture content (Gravmoist), organic matter content (OM), pH, and the (NO_2_^−^ + NO_3_^−^)-N-, K-, Ni-, and P-content (respectively NOx, K, Ni, P), the gene copy numbers of 16S, *pmoA* total (*pmoA*-tot), *pmoA* type Ib (*pmoA*-Ib), and *mcrA*, and the agricultural management variables organic treatment (Oa for organic-amended, Un for unamended) and agricultural practice (NT for no-tillage, Til for tillage). The methane uptake potential is based on the methane uptake rates and lag phases in incubations with ~10 ppm_*v*_ CH_4_ (Ratelow and Laglow, respectively) and ~ 10 000 ppm_*v*_ CH_4_ (Ratehigh and Laghigh, respectively), the methanotrophic community is based on the species abundance of *Methylocaldum szegediense* (M.sze), Upland Soil Cluster α (USCα), *Methylocaldum tepidum* (M.tep), *Methylocystis hirsuta* (M.hir), *Methylocaldum marinum* (M.mar), *Methylosinus trichosporium* (M.tri), Upland Soil Cluster γ (USCγ), *Methylomagnum ishizawai* (M.ish), *Methylocystis parvus* (M.par), and *Methylocystis rosea* (M.ros), and the methanogenic community is based on the abundance of the methanogenic genera.

To explore factors that explain the variance in the methane uptake potential of agricultural soils, an RDA was performed, indicating among others that the pH, (NO_2_^-^ + NO_3_^−^)-N, and moisture content were key governing factors. More specifically, the RDA revealed that the organic fertilization treatment and tillage practice were well-separated into different quadrants, and resulted in RDA1 and RDA2 axes explaining respectively 68.2% and 14.5% of the data variance (Fig. 5A). Furthermore, a permutation test revealed that high methane uptake rates and short lag phases were significantly associated with both organic-amended and no-tilled soils. Also, higher total 16S rRNA, *mcrA*, *pmoA*, and *pmoA* type Ib gene copy numbers correlated negatively with the potential methane uptake rates and positively with longer lag phases at both CH_4_ concentrations. High (NO_2_^−^ + NO_3_^−^)-N content correlated negatively with the potential methane uptake rates at near-atmospheric CH_4_ concentrations, while pH correlated positively with the rates at high CH_4_ concentrations, and the moisture content correlated positively with the rates at both concentrations (Fig. 5A, Table S10).

**Figure 6:**
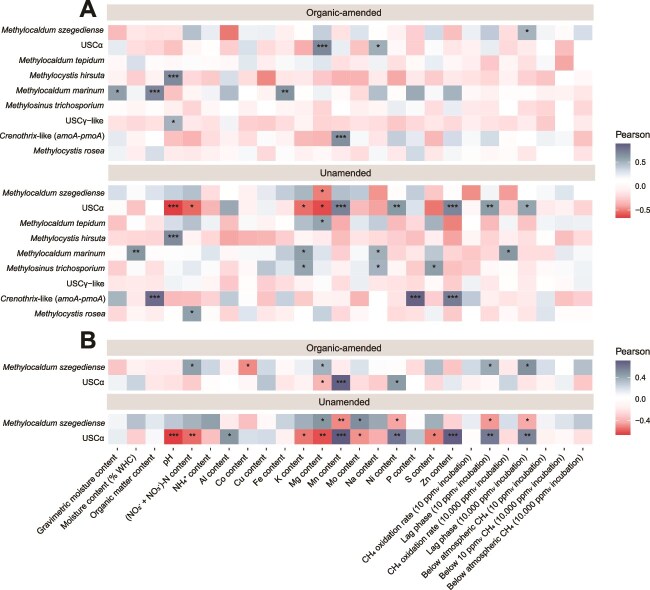
Pearson correlation coefficient matrix of environmental and methane uptake potential variables with the relative abundance of methanotrophic species (A), and with significant differentially abundant methanotrophic species (B), for both organic-amended and unamended agricultural soils (0–15 cm depth).

Next, RDAs were performed to analyze potential controlling factors of the methanotrophic (Fig. 5B) and methanogenic communities (Fig. 5C). The RDA as applied to the methanotrophic community composition, resulted in RDA1 and RDA2 axes explaining respectively 48.8% and 10.9% of the data variance, and the RDA applied to the methanogenic community composition resulted in RDA1 and RDA2 axes explaining respectively 29.3% and 16.3%. The methanotrophic *Methylocaldum* species *M. szegediense*, *Methylocaldum marinum* and *Methylocaldum tepidum* clustered together on the RDA1 axis, maximally opposite of USCα. The *Methylocystis* species clustered together with fellow type II methanotroph *M. trichosporium*. The most abundant methanotroph *M. szegediense* (Fig. 3A) was highly associated with organic-amended soils, and conversely, the second-most abundant methanotroph USCα was associated with unamended soils. A higher pH, moisture, (NO_2_^−^ + NO_3_^−^)-N- and K-content associated with both the organic and no-tillage treatment and were positively correlated with the *Methylocaldum* species cluster (Fig. 5B, Table S10). The relative abundance of *M. szegediense* in organic-amended soils and USCα in unamended soils correlated positively with a longer lag phase at high CH_4_ concentrations. Notably, the differential abundance of *M. szegediense* correlated positively with a longer lag phase at both near-atmospheric and high CH_4_ concentrations in organic-amended soils, and negatively in unamended soils, while the differential abundance of USCα correlated inversely to *M. szegediense*, albeit nonsignificantly in organic-amended soils (Fig. 6). The most abundant methanogenic genus *Methanosarcina* was associated with both organic-amended and tilled soils, while the second-most abundant *Methanocalculus* was associated with both unamended and no-tilled soils. The effect of the organic treatment and agricultural practice on the methanogenic community was thus different from the effect on both the methane uptake potential and the methanotrophic community of the soil. The abundance of *Methanosarcina* correlated positively with the pH, and negatively with the moisture, organic matter, Ni-, and P-content, while these factors correlated inversely with the abundance of *Methanocalculus* (Fig. 5C, Table S10). An additional analysis of the controlling factors of the methane uptake potential, methanotrophic, and methanogenic communities of the upper-top and sub-top layers is provided in the Supplemental results (Fig. S14 and S15).

## Discussion

In this study, the soil CH_4_ uptake potential and the methane cycling microbial communities of agricultural soils under different agricultural management strategies were assessed, and their potential governing factors were determined. Firstly, organic-amendment application can improve the CH_4_ uptake potential of agricultural soils (Fig. 1 and Table S5), and high potential CH_4_ uptake rates and short lag phases were associated with organic-amended soils (Fig. 5A). Secondly, the methanotrophic and methanogenic microbial communities of these soils were unequivocally altered as a result of organic amendment application. Organic-amended soils had, among others, higher *pmoA* and *mcrA* copy numbers (Fig. 2), lower community diversity (Figs 3, 4, Table S8), and a higher differential abundance of *M. szegediense* and *M. horonobensis* (Table S9). Both *M. szegediense* and *M. horonobensis* are the dominant indigenous methane cycling microbes of highly similar green composts as used in this study [27]. Altogether, these results show that organic amendments like compost may improve the soil’s CH_4_ uptake potential, and suggest that compost can serve as a vector for the introduction of methane cycling microbes in agricultural soils.

The CH_4_ uptake potential of organic-amended agricultural soils was improved compared to unamended soils (Fig. 1, Table S5), and based on multivariate analysis, the (NO_2_^−^ + NO_3_^−^)-N content, pH and moisture content were indicated as potential key governing soil factors (Fig. 5A). NO_3_^−^ is a known strong non-competitive inhibitor of oxidation in upland soils, often in combination with a lower pH, attributed to osmotic effects and the NO_3_^−^ induced increase in NH_4_^+^ concentrations, which is a known strong competitive inhibitor of the MMO of MOB [62–65], although NH_4_^+^ itself was not identified as a potential governing factor in these soils. NO_3_^−^ has also been reported to have direct inhibitory effects on the CH_4_ uptake [66, 67]. Considering the soil moisture content as a potential governing factor of CH_4_ uptake, atmospheric CH_4_ needs to diffuse into the soil through the soil pore structure, but when the pore space is filled with water, this diffusion is hampered [68–71]. Next, the atmospheric CH_4_ needs to dissolve into the extracellular water film of MOB cells for it to be available for them to oxidize [72]. Moreover, it was recently shown that high affinity CH_4_ oxidation at atmospheric concentrations is displayed by methanotrophic cultures when in direct contact with air [28]. Altogether, the optimal soil moisture dynamics for atmospheric CH_4_ oxidation is thus a nuanced balance. Hence, the association of high soil moisture content with higher CH_4_ oxidation rates (Fig. 5A) is likely due to a co-correlation with the organic amendment treatment, as the water retention in organic-amended soils is improved [31]. Interestingly, the soils with the longest organic fertilization history (15+ years; Melle and Zürich soils; Table 1), had a significantly higher organic matter and (trace) metal content (Table S4), due to the cumulative effect of organic amendment application [73]. Trace metals like copper, iron, and molybdenum are essential for CH_4_ oxidation by MOB, as their MMO is a metalloenzyme [37, 74], and the high trace metal contents in these soils could have significantly stimulated their potential CH_4_ oxidation rates (Table S10). Overall, the physicochemical effect of the organic amendment application was most pronounced in the top layer of the soil (Supplemental results), signifying a vector function of compost for the introduction of organic matter and trace metals to agricultural soils. Altogether, the results of this study imply that the soil CH_4_ uptake potential is improved by organic amendment application, and it is suggested that this is due to both direct and indirect effects, as we hypothesized. Previously, Ho *et al.* [6] and Brenzinger *et al.* [34] found enhanced CH_4_ uptake rates after organic fertilization of agricultural soils in laboratory mesocosm experiments, confirming the observed positive effect of organic fertilization, and reiterating its potential to contribute to CH_4_ mitigation. However, in a previous study executed in parallel at the same field sites as this study, the *in situ* field CH_4_ fluxes showed no effect of organic fertilization [42]. It was hypothesized that a potential *in situ* effect was masked due to the fluctuating nature of daily soil CH_4_ fluxes and environmental conditions in field situations [42, 75]. Compared to other studies, the observed potential CH_4_ oxidation rates at near-atmospheric and high CH_4_ concentrations both fell in the upper end of the reported range for agricultural soils (Table S11), highlighting the CH_4_ sink potential of these agricultural soils.

The methanotrophic and methanogenic microbial communities of agricultural soils were significantly altered as a result of organic amendment application. Both communities increased in size, but their diversity decreased. More importantly, in organic-amended soils the methanogenic and methanotrophic communities became dominated by single species, respectively *M. horonobensis* and type Ib MOB *M. szegediense*, the latter at most locations to the detriment of USCα (Fig. 4A, B, and Table S9). Recent work has shown that a decrease in the complexity of soil microbial communities due to microbial inoculants is accompanied by an increase in stability [41], suggesting that the introduced methane cycling microbes via compost could become stable members of the soil’s microbial communities. The methanotrophic community was shaped by the (NO_2_^−^ + NO_3_^−^)-N content, pH and moisture content, which are all well-known important biogeochemical shaping factors [16, 76]. Furthermore, it has recently been determined that the methanotrophic and methanogenic communities of green composts are hyper-dominated by these same species, *M. szegediense* and *M. horonobensis*, with relative abundances up to 100% [27]. The green composts used for organic fertilization in this study were highly similar to the green composts analyzed by van den Bergh *et al.* [27] in terms of initial substrate, production method, and key characteristics (Table S12), thereby strongly suggesting that *M. szegediense* and *M. horonobensis* were also the dominant indigenous methane cycling microbes of the composts used in this study. Taken together, this would advocate for a vector function for compost for the introduction of these methane cycling microbes. Previously, the introduction of methanogens via compost has been shown on a small scale [40], but the results of our study clearly show that compost amendment can modify biogeochemically relevant microbial communities substantially also at field scale.

It should be noted however that this study is subject to some limitations related to site variability, as the included study sites differed in soil properties, organic amendment application history, plant cover, and seasonality (Table 1). As such, the results of this study should be carefully extrapolated to broader conclusions. Also, the potential gaps arising by the upscaling of laboratory experiments to field-scale functioning should be taken into account [77]. Our rationale for site selection was based on the availability of existing long-term field experiments with an organic fertilization treatment, as this allowed for an extensive screening of the effect of compost application on the CH_4_ uptake potential and methane cycling microbial communities. While the CH_4_ uptake rates and corresponding lag phases were only significantly improved in some sites, clear significant stimulatory trends were observed across all sites, that mostly were site-independent, as indicated by the nonsignificant interaction between organic treatment and study site. And although the effects on the methane cycling communities of soils appeared to be sometimes driven by the effects at individual locations, robust differential abundance analyses still indicated clear effects of organic fertilization on the methane cycling microbial communities. So, the results of this study point in the direction of distinct and consistent effects on the soil CH_4_ uptake potential and involved microbial communities, but these should be interpret in view of context- and site-dependency, thereby allowing for careful generalizations and interpretations about the CH_4_ mitigation potential of organic fertilization.

The emergence as the dominant members within the methanotrophic and methanogenic communities of agricultural soils by methane cycling microbes introduced via compost implies that these microbes increase in biomass after compost application. But so far, no *Methylocaldum* species has been demonstrated to oxidize at near-atmospheric concentrations [78, 79]. Recently however, using a membrane-based cultivation method and after a 6-month adaptation period, conventional MOB species have been shown to oxidize CH_4_ at atmospheric concentrations by gearing down their C-assimilation and diverting all available energy to CH_4_ oxidation machinery [28]. Possibly, *Methylocaldum* species are also capable hereof when cultivated as such, which should be a focus of future research. Furthermore, conventional MOB are capable of oxidizing CH_4_ to (sub-)atmospheric concentrations after activation with high CH_4_ concentrations [19]. It can thus also be speculated that the compost-originating *M. szegediense* are highly activated during the composting process, using the energy stored therein to oxidize CH_4_ at low concentrations, incorporating it in their biomass and thereby increasing their abundance in soil. It has been shown before in soil systems that under high CH_4_ conditions the methanotrophic community is dominated by *Methylocaldum* species [80]. When their energy reserves are depleted, the resilient *M. szegediense* then remains dominantly abundant in soil, with limited to no atmospheric CH_4_ uptake activity, which would explain the transient nature of the stimulatory effect Ho *et al.* [6, 24] had observed. The negative correlation between total and type Ib *pmoA* copy numbers and the CH_4_ uptake potential (Figs 5A and 6B) further supports this, suggesting that these MOB are not responsible for the observed enhanced atmospheric CH_4_ uptake. Considering this transient effect, the improvement of the CH_4_ uptake potential was most apparent in the two soils with the shortest time since organic amendment application (Melle and Zürich; Table 1). Therefore, future research should further investigate such a possible time effect by means of a time-controlled series analysis. Furthermore, the phylum *Methylomirabilota*, previously known as NC10 and containing the nitrite-dependent anaerobic methane oxidizing (N-DAMO) bacterial lineages, was observed in low relative abundances (<3.0%) in all soils (Fig. S7). However, N-DAMO is severely limited by oxygen exposure [81, 82], and is therefore not a likely contributor to the observed CH_4_ uptake in these oxic soils. Also, the high-affinity USCα, which are vulnerable to perturbation by agricultural practices like tillage and fertilization [8, 24], were substantially reduced in abundance in organic-amended soils (Fig. 4, Table S9) and were associated with a longer lag phase for the oxidation of CH_4_ (Fig. 6B). Altogether, this suggests that USCα are also not responsible for the improved CH_4_ uptake potential, and raises the question which MOB in fact are?

It could be speculated that conventional type IIa MOB, which includes the genera *Methylocystis* and *Methylosinus*, are responsible for the observed enhanced atmospheric CH_4_ uptake in organic-amended agricultural soils, as is also supported by the high type II MOB copy numbers (Fig. 2, S3). Type IIa *Methylocystis* sp. strains are capable of oxidizing CH_4_ at circum-atmospheric concentrations, while recently it was shown that *M. trichosporium* lacks this capacity [28, 78]. In organic-amended soils, Ho *et al.* [24] found ^13^C-enriched PLFA profiles belonging to type IIa MOB, in incubations at 40 ppm*_v_* CH_4_. In this study, multiple *Methylocystis* species were differentially more abundant in organic-amended soils (Table S9) and could thus be likely candidates. Future research should focus if these type IIa MOB are indeed active at atmospheric CH_4_ concentrations in agricultural soils using PLFA-SIP or cell-labelling approaches that allow single-cell identification [21], and if the use of organic amendments like compost indeed improves their CH_4_ uptake potential. Also, engineering organic amendments towards desired stimulating effects on CH_4_ mitigation, as recently described by Chavez-Rico *et al.* [83], should be considered.

In conclusion, this study showed that organic fertilization with compost improves the CH_4_ uptake potential of the soil and drastically alters the methane cycling microbial community of agricultural soils by introducing methanotrophic and methanogenic microbes via the compost. Key implication hereof is that compost could be used to steer the methane cycling or other relevant microbial communities of agricultural soils, thereby contributing to CH_4_ mitigation and shift towards climate-smart soils.

## Supplementary Material

Supplemental_material_ycaf139

Supplemental_tables_ycaf139

## Data Availability

Sequencing data can be found at the European Nucleotide Archive (ENA) under the accession number PRJEB75729 (http://www.ebi.ac.uk/ena/data/view/PRJEB75729). Further datasets generated and/or analyzed during this study are available via doi:10.5061/dryad.dz08kps8h.
