# Supplementary material for: Improved methane mitigation potential and modulated methane cycling microbial communities in arable soil by compost addition
Source: ISME Commun. 2025 May 29;5(1):ycaf139. doi: 10.1093/ismeco/ycaf139 (PMC12395338; doi:10.1093/ismeco/ycaf139)
Supplement: Supplemental_material_ycaf139 [file supplemental_material_ycaf139.pdf]

Improved methane mitigation potential and modulated methane cycling microbial communities in arable soil by compost addition

Stijn G. van den Bergh, Iris Chardon, Marion Meima-Franke, Germán Pérez, Gabriel S. Rocha, Kristof Brenzinger, Gerard W. Korthals, Jochen Mayer, Mathias Cougnon, Dirk Reheul, Wietse de Boer, Paul L.E. Bodelier

ISME Communications, 2025

1. Supplemental material & methods

1.1. Laser diffraction analysis of the particle size distribution

Soil samples from the field sites of this study, collected as described previously [1], were used for laser diffraction analysis (LDA) of the soil particle size distribution (PSD). The samples were analyzed without the chemical removal of organic matter, calcium carbonate and other ionic compounds like ferrous oxides among others [2, 3]. The PSD was determined using a Mastersizer 3000 Laser Diffraction Analyzer equipped with a Hydro LV Dispersion Unit (Malvern Panalytical, Malvern, United Kingdom). The refractive data was analyzed using the Mastersizer software (version 3.81) (Malvern Panalytical), using the 'general purpose' model for 'non-spherical particles'. The calculation of volume (%) was based on the Mie theory [4], using a refractive index of 1.52, an absorption coefficient of 0.1, and a dispersant refractive index of 1.33 [2, 5, 6]. Demineralized water was used as dispersant and stirrer speed was set at 2300 rpm. Three technical replicates per sample were measured, and approximately 300 mg (clay-rich soils) or 1000 mg (sandy soils) per replicate was loaded in the dispersion unit. Samples were dispersed by sonicating for 3 min at 50% power [7], to achieve an ideal obscuration of 15-20%. After a cooldown period of 30 seconds after sonication, the PSD was determined as the average of 3 measurements, each consisting of

measuring diffraction for 20 seconds with a red light source (max. 4mW He-Ne, 632.8nm) and subsequently 10 seconds with a blue light source (max. 10mW LED, 470nm). Refractive data that did not comply with the Malvern quality control guidelines or with an obscuration <10% or >25% were discarded, and all transformed raw data were exported to Microsoft Excel. As defined by Bieganowski et al. [2], the PSD on basis of volume %, was divided into sand, silt and clay fractions using 60.3 μm as the sand-silt boundary and 8 μm as the silt-clay boundary, and subsequently classified according to the USDA soil texture triangle [8].

1.2. DNA extraction and qPCR assays

Each qPCR assay was performed in duplicate, and each qPCR reaction was prepared by a QIAgility PCR setup system (Qiagen, Venlo, The Netherlands), consisting of 2x SensiFAST SYBR (BIOLINE, Alphen aan den Rijn, The Netherlands) for *pmoA* and *mcrA*, or iTaq Universal SYBR Green Supermix (Bio-Rad Laboratories, Lunteren, The Netherlands) for 16S, forward and reverse primers (Integrated DNA Technologies, Leuven, Belgium), bovine serum albumin (5 μg μL^{-1}; Invitrogen, Breda, The Netherlands), diluted template DNA (10 ng μL^{-1}), and optionally nuclease-free water. Amplicon specificity was inferred from the melt curve.

1.3. Statistical analyses

All statistical analyses were done using R (version 4.3.2) [9]. The mean methane oxidation rates, lag phases, physicochemical properties, and gene abundances were tested for normality by Shapiro-Wilk test and for homogeneity of variance by Levene's test, and log transformed if necessary. To assess the effect of the organic amendment application per location, paired t-tests were performed. Treatment, location, and

practice effects, and the interaction of the latter two with the organic treatment, were assessed using two-way ANOVA, Scheirer-Ray-Hare, or χ^2-tests. Pairwise comparison of soil layers was performed using Tukey’s HSD, Dunn’s or least-square means *post-hoc* tests.

Multivariate analyses were performed using the *vegan* package in R [10]. A redundancy analysis (RDA) was performed to determine controlling factors of methane uptake rates, using the variables treatment, practice, all 16S, *pmoA* (total, type Ib, and type II), and *mcrA* gene copy numbers, and all available physicochemical variables. To analyze methanotrophic and methanogenic community composition-environment relationships on the species and genus level, respectively, initial detrended correspondence analyses (DCA) were performed. DCA1 axes lengths of <3.0 indicated linear community responses, and thus subsequent RDAs were performed, using the variables treatment, practice, and all available physicochemical variables. Environmental variables significantly explaining the observed variance were identified by a Monte Carlo permutation test (999 unrestricted permutations).

To assess the effect of the organic treatment on the total bacterial, methanotrophic, and methanogenic community compositions, a PERMANOVA test was performed based on Bray-Curtis dissimilarity matrices using the *vegan* [10] and *microeco* [11] packages. A Pearson correlation analysis between the relative and differential abundances of methanotrophic species and environmental variables was performed using the *microeco* package [11].

2. Supplemental results

2.1. Physicochemical properties

Additional to the described results in the main paper, organic-amended soils (0-15 cm depth) overall had a lower Ni content than unamended soils (Scheirer-Ray-Hare test, $p \leq 0.05$) (Table S5). Considering the comparison of the physicochemical properties between organic-amended and unamended soils within one location (Table S4), there were no differences in any of the physicochemical properties of Vredepeel (both no-tilled and tilled), Valthermond and Lelystad soils. Organic-amended Melle soil had a higher pH, higher gravimetric moisture, organic matter and Fe content, and a lower Na content, and organic-amended Wageningen soil had a higher Cu and S content than their respective unamended counterparts. The organic-amended Zürich soil was most distinctly different from its unamended counterpart, as it had a higher moisture and organic matter content, pH, and (NO_2^- + NO_3^-)-N, Mg and P content, and concurrently a lower metal (Al, Cu, Fe, Mn, Ni and Zn) content than the unamended Zürich soil (Table S4).

The organic-amended upper-top soil layers (0-7.5 cm depth) overall had higher K (ANOVA, $p \leq 0.01$), Mo and P contents (Scheirer-Ray-Hare test, both $p \leq 0.05$), although Mo was not detected in both Wageningen and Zürich soils (Table S5). Furthermore, regarding the comparison of the physicochemical properties between organic-amended and unamended soils within one location, the effect of the organic was more apparent in this upper-top layer of the soil than in the other soil layers (Table S4). No-tilled organic-amended Vredepeel upper-top soil had a higher Mn and Mo content, organic-amended Valthermond upper-top soil had a higher Na content, and organic-amended Lelystad upper-top soil had a higher Mg, Na, P, and S content than their respective unamended counterparts. Organic-amended Melle upper-top soil though,

had a lower Mn, Na, and S content than the unamended Melle upper-top soil, and higher gravimetric moisture and organic matter contents. Lastly, organic-amended Wageningen upper-top soil had a higher S content, and organic-amended Zürich upper-top soil had a higher moisture and organic matter content, pH, K, Mg and S content, and a lower Al, Mn, Ni and Zn content, than the respective unamended counterparts (Table S4).

Organic-amended sub-top soil layers (7.5-15 cm depth) had higher P content (ANOVA, $p \leq 0.05$) than their unamended counterpart. There were no further overall treatment or practice effects on the physicochemical properties of the sub-top soil layer (Table S5). Furthermore, considering the comparison of the physicochemical properties between organic-amended and unamended soils within one location for the sub-top layer (Table S4), organic-amended Vredepeel sub-top soil (no-tilled) had a higher Cu content, and organic-amended Lelystad sub-top soil had a lower pH, and a higher (NO_2^- + NO_3^-)-N, Na and S content than the unamended sub-top soil. The organic-amended Zürich sub-top soil was again the most distinctly different from its unamended counterpart, as it had a higher moisture and organic matter content, pH, and Mg and P content, and a lower Al, Cu, Mn, Na, Ni and Zn content (Table S4).

Lastly, a pairwise comparison between the soil and its upper-top and sub-top layers showed that the sub-top layer had significantly lower K, Mn and P content than the upper-top layer (Dunn's test, $p \leq 0.001$, $p \leq 0.05$, and $p \leq 0.05$, respectively) (Table S5).

2.2. Laser diffraction analysis of the particle size distribution

Laser diffraction analysis (LDA) was used to assess the particle size distribution (PSD) (Table S6). The PSD of organic-amended Vredepeel soil was classified as sandy loam, and of the unamended Vredepeel soil as loamy sand for both the no-tilled and tilled

soils. For all other individual locations, the organic-amended and the unamended soils had the same classification: Valthermond and Wageningen soils had a PSD classified as sandy loam, Lelystad and Zürich soils as loam, and Melle soil as silt loam.

The emergence of the contemporary laser diffraction analysis of physical soil characteristics, such as the soil aggregate stability among others, can overcome the limitations of traditional methods like sieve-pipetting [1, 12–14]. In this study, the LDA of the PSD was a reliable method regarding the repeatability and high-throughput sample handling. However, the LDA output in volume% differed substantially from the traditional weight-based PSD determination (Table 1 & S6), even despite the silt-clay cut-off transformation. This is because LDA underestimated the clay fraction, which is in accordance with recent studies [2, 15]. To harmonize LDA with traditional PSD classification methods, we suggest expanding the optimization of their compatibility by wet-sieving the sand fraction [3, 15], transformation of the clay-silt cutoff [2], and the use of pedotransfer functions to correct for chemical soil properties [5, 15]. Similar to the approach of Makó et al. [5], country- or region-specific pedotransfer functions, accounting for scale-specific soil properties, should be developed for optimal conversion of LDA data.

2.3. Methane uptake potential of agricultural soils

Taking all data together, the effect of the organic fertilization was most evident in the Zürich soil, as in both incubations with near-atmospheric and high methane concentrations, the potential methane uptake rates were significantly higher and lag phases significantly shorter for the organic-amended soil (0-15 cm depth) than in its unamended counterpart. This also applied for the organic-amended upper-top (0-7.5 cm depth) and sub-top (7.5-15 cm depth) layers of Zürich soil (Table S5).

Furthermore, considering the comparison of the CH_4 uptake potential in the near-atmospheric CH_4 incubation (~10 ppm$_v$) between organic-amended and unamended soils within one location, the potential methane oxidation rate was higher in organic-amended Melle soil (Figure 1) and its upper-top (Figure S1) and sub-top layers (Figure S2) than in their unamended counterparts. Also, the organic-amended Wageningen upper-top layer had a shorter lag phase than the unamended upper-top layer (Figure S1). Notably, the upper-top layer of tilled soils had a better potential to oxidize to sub-atmospheric CH_4 concentrations than the upper-top layer of no-tilled soils (χ^2-test, $p \leq 0.05$) in the near-atmospheric CH_4 incubation (Table S5).

Regarding the comparison of the CH_4 uptake potential in the high CH_4 incubation (~10,000 ppm$_v$) between organic-amended and unamended soils within one location, the effect of the organic amendment application was most evident in the upper-top layer of the soil, as in the organic-amended Valthermond, Melle, and Zürich upper-top soil layers the potential methane uptake rate was improved compared to the unamended upper-top soil layers, and in organic-amended Valthermond, Lelystad, Melle, and Zürich upper-top soil layers the lag phase was lower as well (Figure S1).

2.4. Abundance analyses of methane cycling bacteria and Archaea

Regarding the comparison of the abundance of the total bacterial community between organic-amended and unamended soils within one location, there were no soils (0-15 cm depth) of individual locations with a significantly higher or lower bacterial 16S rRNA copy number as a result of organic amendment application (Figure 2), even though the bacterial 16S rRNA copy number was overall higher in organic-amended soils than in unamended soils. The same effect was observed for the upper-top (0-7.5 cm depth; Figure S4) and sub-top (7.5-15 cm depth; Figure S5) layers. Notably however, the

upper-top layer of tilled soils had a lower bacterial 16S rRNA copy number than the upper-top layer of no-tilled soils (ANOVA, $p \leq 0.01$) (Figure S4 & Table S5).

Considering the comparison of the abundance of methanogens between organic-amended and unamended soils within one location, this was increased in organic-amended Vredepeel (both tilled and no-tilled) and Melle soil, per the *mcrA* copy numbers (Figure 2). In the soil's upper-top layer, the same significant effect was found at Vredepeel (tilled) and Melle (Figure S4), whilst in the soil's sub-top layer the *mcrA* copy number was increased in organic-amended Valthermond and Melle soil (Figure S5). Also, the sub-top layer of tilled soils had a higher *mcrA* gene copy number than that of no-tilled soil (Scheirer-Ray-Hare test, $p \leq 0.05$) (Figure S5 & Table S5).

Regarding the comparison of the abundance of methanotrophs between organic-amended and unamended soils within one location, the total *pmoA* copy number was higher in organic-amended Melle soil (Figure 2) and its upper-top soil layer (Figure S4). Furthermore, the organic-amended sub-top layer of Zürich soil had a higher total *pmoA* copy number than the unamended sub-top layer (Figure S5). Type Ib *pmoA* copy numbers were higher in organic-amended Melle and no-tilled Vredepeel soils (0-15 cm depth; Figure 2), and in the upper-top and sub-top layer of organic-amended Melle and tilled Vredepeel soils (Figure S4 & S5).

2.5. Total bacterial, methanotrophic, and methanogenic community analysis

The total bacterial community of the upper-top (0-7.5 cm depth) and sub-top (7.5-15 cm depth) layers of the soil (Figure S8 & S9) was to a large extent similar to the total bacterial community of the soil (0-15 cm depth) as described in the main text (Figure S7). The phylum *Proteobacteria* also had a relative abundance of around 10 to 20% in the upper-top layer of the soil at all locations, and in the sub-top layer of the soil at all

locations except for Wageningen (5 to 10%). The phylum *Methylomirabilota* had a relative abundance between 0.1 and 3.6% in both upper-top and sub-top layers at all locations. Furthermore, the notable high relative abundance of *Firmicutes* in the Wageningen soil was driven by a high relative abundance in the sub-top layer rather than the upper-top layer. Overall, the alpha diversity of the total bacterial community in both the upper-top and sub-top layers were not affected by the use of organic amendments, as was also the case for all individual locations in both soil layers. The beta diversity of the total bacterial community was in the upper-top and sub-top layers of the soil overall not significantly different between organic- and unamended soils. In the upper-top layer of Valthermond and Zürich soil however, there was a significant difference in the beta diversity between the total bacterial communities of organic- and unamended soils (PERMANOVA, $p \leq 0.05$ and $p \leq 0.01$, respectively). In the sub-top layer of the soil, only Zürich soil had significantly different beta diversity as a result of the organic treatment (PERMANOVA, $p \leq 0.01$) (Table S8).

Considering the methanotrophic communities of the upper-top (Figure S10) and sub-top layer (Figure S11), these were both dominated by *Methylocaldum szegediense*, which had a higher relative abundance in organic-amended soils than in unamended soils in both layers (ANOVA, both $p \leq 0.001$). In the upper-top layer, *Methylocaldum marinum* had a higher relative abundance in unamended soils (ANOVA, $p \leq 0.01$), and in the sub-top layer, USCα, *M. marinum,* and TUSC had a higher relative abundance in unamended soils (ANOVA, all $p \leq 0.05$). Overall, as revealed by a Random Forest analysis, in the upper-top layer, *M. szegediense*, TUSC, *Methylomagnum ishizawai*, *Methylocystis echinoides*, and cluster RA21-like methanotrophs had a higher differential abundance in organic-amended soils. In the sub-top layer, *M. szegediense* and *Skermanella aerolata* had a higher differential abundance in organic-amended

soils (Table S9). In both the upper-top and sub-top layer of the soil, the alpha diversity of the methanotrophic community was lower in organic-amended soils than in unamended soils (ANOVA, both $p \leq 0.001$). Considering the alpha diversity at individual locations, in the upper-top layer, the alpha diversity was significantly lower in the organic-amended soil at four locations (Vredepeel, Valthermond, Melle and Zürich), whereas in the sub-top layer this was at two locations (Vredepeel and Zürich). The beta diversity was in both layers also significantly different between organic- and unamended soils (PERMANOVA, both $p \leq 0.01$), and, again, in the upper-top layer (four individual locations; Vredepeel, Valthermond, Melle and Zürich) this effect was more pronounced than in the sub-top layer (three individual locations; Vredepeel, Valthermond and Zürich) (Table S8).

Regarding the methanogenic communities of the upper-top and sub-top layers of the soil (Figure S12 & S13), these were almost identical to the methanogenic community of the soil (0-15 cm depth) as described in the main text (Figure 4). The methanogenic communities were dominated by the genera *Methanosarcina*, *Methanocalculus*, and *Methanomassiliicoccus*, with *Methanosarcina horonobensis* being the most dominant species. *M. horonobensis* had a higher relative abundance in the organic-amended soil compared to the unamended soil in both the upper-top and sub-top layer (ANOVA, both $p \leq 0.001$). In the upper-top layer, *Methanosarcina spelaei* had a higher relative abundance in unamended soil (ANOVA, $p \leq 0.05$), and in the sub-top layer both *Methanosarcina mazei* and *Methanobrevibacter millerae* had a higher relative abundance in unamended soil (ANOVA, both $p \leq 0.05$). In both soil layers, *M. horonobensis* had a higher differential abundance in organic-amended soil (Random Forest, both $p \leq 0.001$), and in the upper-top layer *Methanosarcina barkeri* also had a higher differential abundance in the organic-amended soil (Table S9). The overall

alpha diversity of the methanogenic community was in both the upper-top and sub-top layers of the soil lower in organic-amended soils than in unamended soils (ANOVA, $p \leq 0.01$ and $p \leq 0.05$, respectively). Also, the beta diversity was in both soil layers significantly different between the organic-amended soil and in its unamended counterpart (PERMANOVA, both $p \leq 0.01$). Similar to the effect of the use of organic amendments on the alpha and beta diversity of the methanotrophic community, the effect on the methanogenic community was more apparent in the upper-top layer of the soil. In the upper-top layer, the alpha diversity was significantly lower in no-tilled Vredepeel, Valthermond, Melle, and Zürich soils, and the beta diversity was significantly different in Vredepeel (both no-tilled and tilled), Valthermond, Melle, and Zürich soils. In the sub-top layer of the soil, only organic-amended Zürich soil had a significantly lower alpha diversity, and Vredepeel, Valthermond, and Zürich soil had a significantly different beta diversity (Table S8).

Overall, the effect of the organic amendment application on the methanotrophic and methanogenic communities was most pronounced in the Zürich soil, where the organic-amended soil had a higher 16S alpha diversity, but lower *pmoA* and *mcrA* alpha diversity. Also, the beta diversity on basis of 16S, *pmoA*, and *mcrA* was significantly different between the organic-amended and unamended Zürich soil and both the upper-top and sub-top soil layers (Table S8).

2.6. Potential controlling factors of the methane uptake potential and associated microbial communities

The factors contributing to the variance observed in the methane uptake potential of the upper-top (0-7.5 cm depth; Figure S14a) and sub-top layer (7.5-15 cm depth; Figure S15a) of the soil were assessed with a redundancy analysis (RDA). It resulted

in RDA1 and RDA2 axes explaining respectively 52.6% and 23.1% of the data variability in the upper-top layer, and respectively 74.4% and 14.2% in the sub-top layer. In the upper-top layer, only the tillage practice was a significantly contributing factor, whilst in the sub-top layer both the organic treatment and the tillage practice were significant factors, as revealed by permutation tests (Table S10). Furthermore, it was revealed that in the upper-top layer 16S and *pmoA* (total, type Ib, and type II) gene copy numbers, pH, Al-, and Fe-content were significantly correlating with the methane uptake potential. In the sub-top layer of the soil *pmoA* (total, type Ib, and type II) and *mcrA* gene copy numbers, pH, moisture content, Fe-, K-, and Mn-content correlated significantly. The clustering of the potential methane uptake rates and lag phases in the RDAs of both the upper-top and sub-top layers was close to identical as in the RDA of the soil (0-15 cm depth), as described in the main text (Figure 5), indicating that in both soil layers similar significant interactions were contributing to the methane uptake potential of the soil.

The controlling factors of the methanotrophic and methanogenic communities of the upper-top (Figure S14bc) and sub-top layer (Figure S15bc) of the soil were also assessed with RDAs. For the methanotrophic community, this resulted in RDA1 and RDA2 axes explaining respectively 53.9% and 8.7% for the upper-top layer, and 53.1% and 11.8% for the sub-top layer. For the methanogenic community, the RDA1 and RDA2 axes explained respectively 26.6% and 15.5% for the upper-top layer, and 22.2% and 14.1% of the sub-top layer. The clustering of the methanotrophic species in the RDA on the methanotrophic community of both soil layers was almost identical to the RDA of the soil as described in the main text (Figure 5b), indicating that in both soil layers similar significant interactions were shaping the methanotrophic community. In both soil layers, the *Methylocaldum* species clustered maximally opposite of USCα

on the RDA1 axis, with the latter being associated with both unamended and tilled soils, and the *Methylocaldum* species with both organic-amended and no-tilled soils. Also, in both soil layers, the pH and (NO_2^-+NO_3^-)-N-content were significantly contributing factors, positively correlating with the *Methylocaldum* sp. cluster, and negatively with USCα (Table S10).

The RDAs also showed similar clustering of the methanogenic community in the upper-top layer of the soil, but notably not in the sub-top layer compared to the RDA of the soil (0-15 cm depth) (Figure 5c, S14c & S15c). Furthermore, only the Fe- and Mg-content were significantly correlating factors in the sub-top layer, whilst in the upper-top layer the pH, organic matter and moisture content, (NO_2^-+NO_3^-)-N-, K-, Mg-, Mn-, and Zn-content were all significantly correlating with the methanogenic community composition. The methanogenic community composition of the sub-top layer can thus be less well explained by these physicochemical factors than the methanogenic community of the soil as described in the main text and the upper-top layer, and is thus possibly shaped by different controlling factors. The most abundant genus *Methanosarcina* was in both soil layers associated with both organic-amended and tilled soils, and the second-most abundant genus *Methanocalculus* was in both soil layers associated with both unamended and no-tilled soils.

3. Supplemental figures

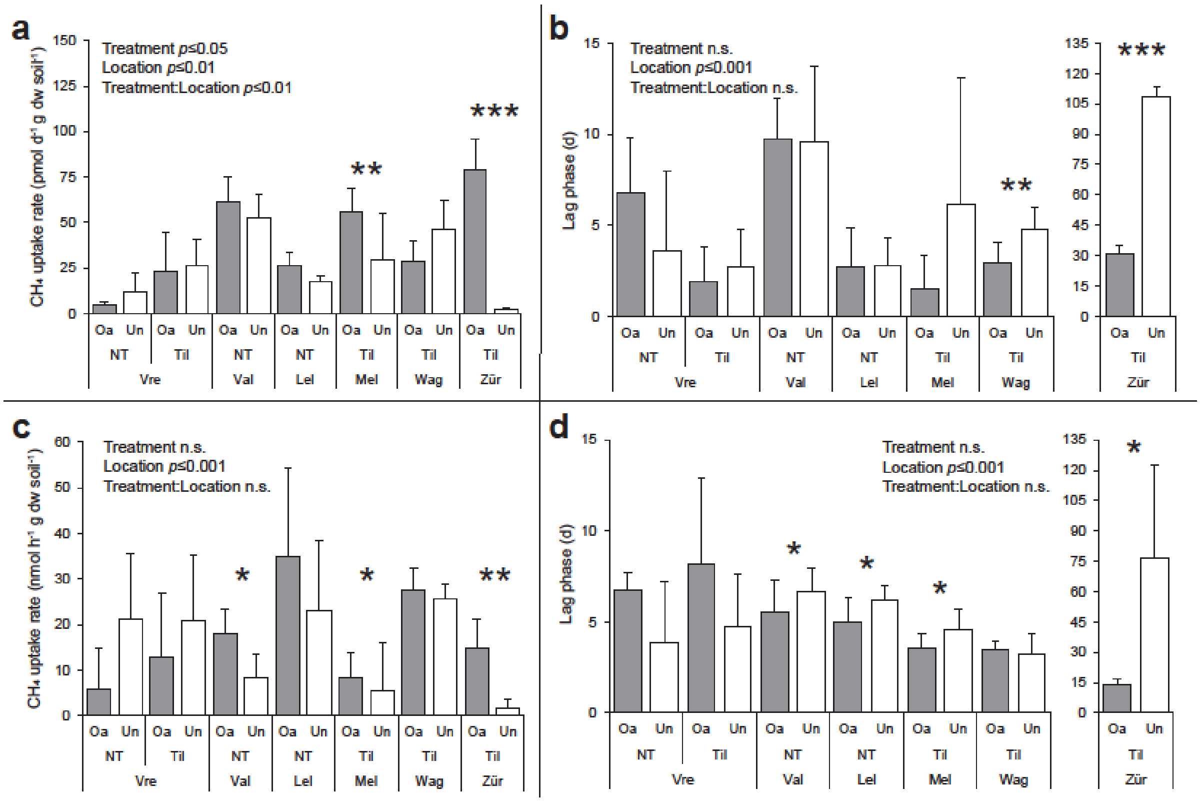

Figure S1. Potential methane uptake rates (a, c) and lag phases (b, d) in incubations with ~10 ppm$_v$ (a, b) and ~10.000 ppm$_v$ (c, d) CH_4 (mean ± SD; *n*=4-6) of the upper-top layer of agricultural soils (0-7.5 cm depth) of visited field sites (Vre – Vredepeel; Val – Valthermond; Lel – Lelystad; Mel – Melle; Wag – Wageningen; Zür – Zürich), varying in organic treatment (Oa – organic-amended; Un – unamended) and agricultural practice (NT – no-tillage; Til – tillage). Significant differences within a location are indicated with an asterisk (paired t-test; * $p≤0.05$; ** $p≤0.01$; *** $p≤0.001$). Treatment and location effects, and their interaction, are given if significant (one-way ANOVA or Scheirer-Ray-Hare test).

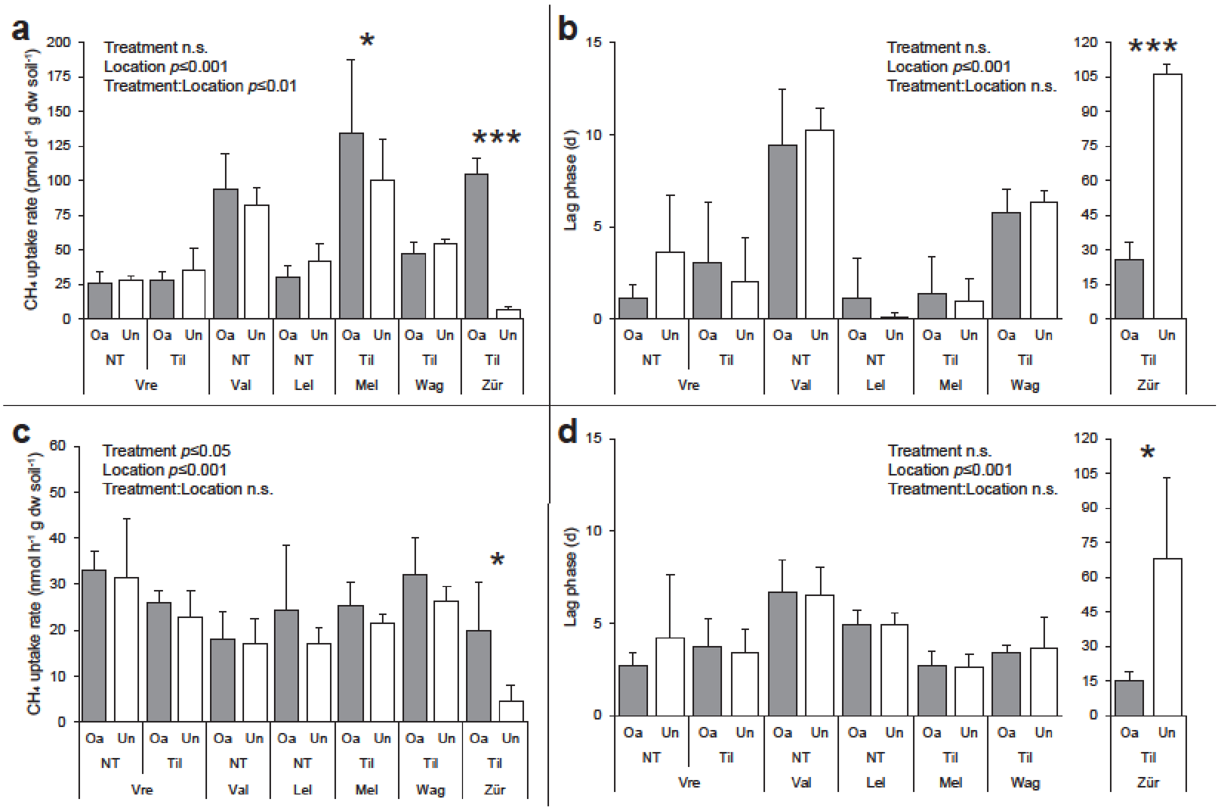

Figure S2. Potential methane uptake rates (a, c) and lag phases (b, d) in incubations with ~10 ppm$_v$ (a, b) and ~10.000 ppm$_v$ (c, d) CH_4 (mean ± SD; n=4-6) of the sub-top layer of agricultural soils (7.5-15 cm depth) of visited field sites (Vre – Vredepeel; Val – Valthermond; Lel – Lelystad; Mel – Melle; Wag – Wageningen; Zür – Zürich), varying in organic treatment (Oa – organic-amended; Un – unamended) and agricultural practice (NT – no-tillage; Til – tillage). Significant differences within a location are indicated with an asterisk (paired t-test; * $p \leq 0.05$; ** $p \leq 0.01$; *** $p \leq 0.001$). Treatment and location effects, and their interaction, are given if significant (one-way ANOVA or Scheirer-Ray-Hare test).

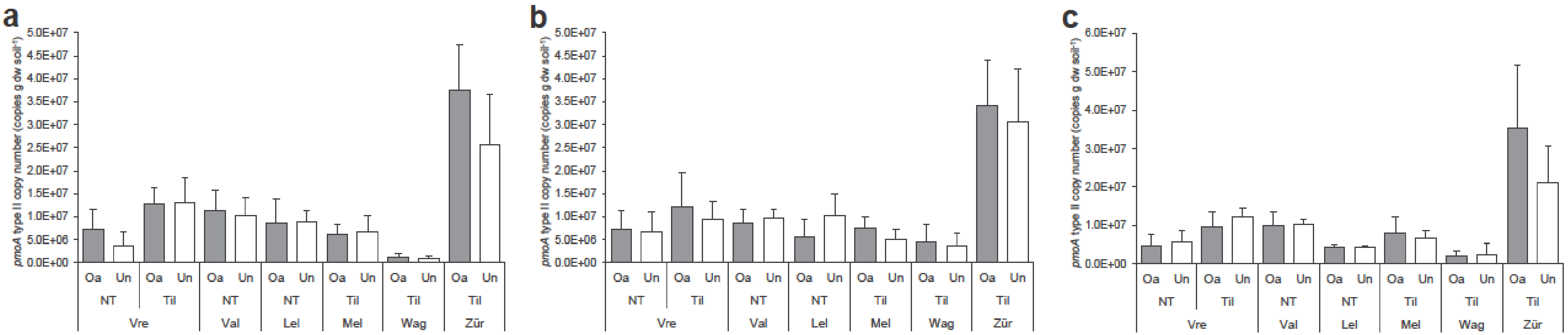

Figure S3. Abundance of *pmoA* type II gene per gram dry weight soil (mean ± SD; *n*=4-6) of agricultural soils (0-15 cm depth) (a), its upper-top layer (0-7.5 cm depth) (b), and its sub-top layer (7.5-15 cm depth) (c) of visited field sites (Vre – Vredepeel; Val – Valthermond; Lel – Lelystad; Mel – Melle; Wag – Wageningen; Zür – Zürich), varying in organic treatment (Oa – organic-amended; Un – unamended) and agricultural practice (NT – no-tillage; Til – tillage). Significant differences within a location are indicated with an asterisk (paired t-test; * p≤0.05; ** p≤0.01; *** p≤0.001). Treatment and practice effects, and their interaction, are given if significant (one-way ANOVA or Scheirer-Ray-Hare test)

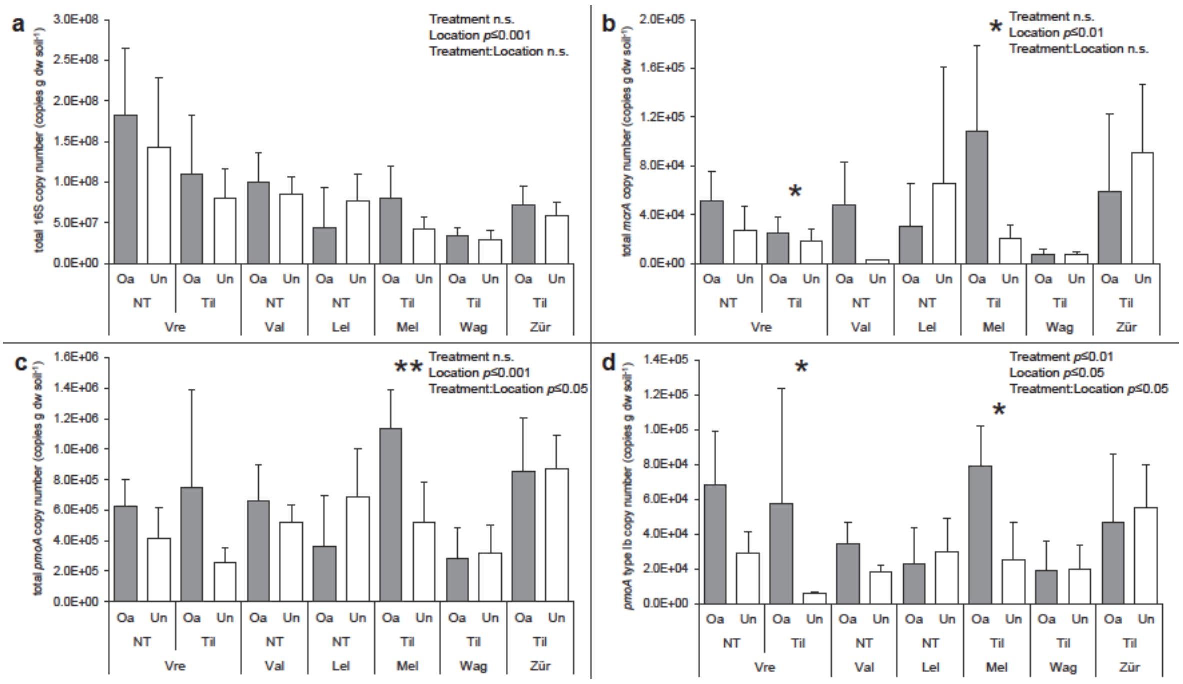

Figure S4. Abundance of 16S (a), *mcrA* (b), total *pmoA* (c), and *pmoA* type Ib (d) genes per gram dry weight soil (mean ± SD; *n*=4-6) of the upper-top layer of agricultural soils (0-7.5 cm depth) of visited field sites (Vre – Vredepeel; Val – Valthermond; Lel – Lelystad; Mel – Melle; Wag – Wageningen; Zür – Zürich), varying in organic treatment (Oa – organic-amended; Un – unamended) and agricultural practice (NT – no-tillage; Til – tillage). Significant differences within a location are indicated with an asterisk (paired t-test; * *p*≤0.05; ** *p*≤0.01; *** *p*≤0.001). Treatment and location effects, and their interaction, are given if significant (one-way ANOVA or Scheirer-Ray-Hare test).

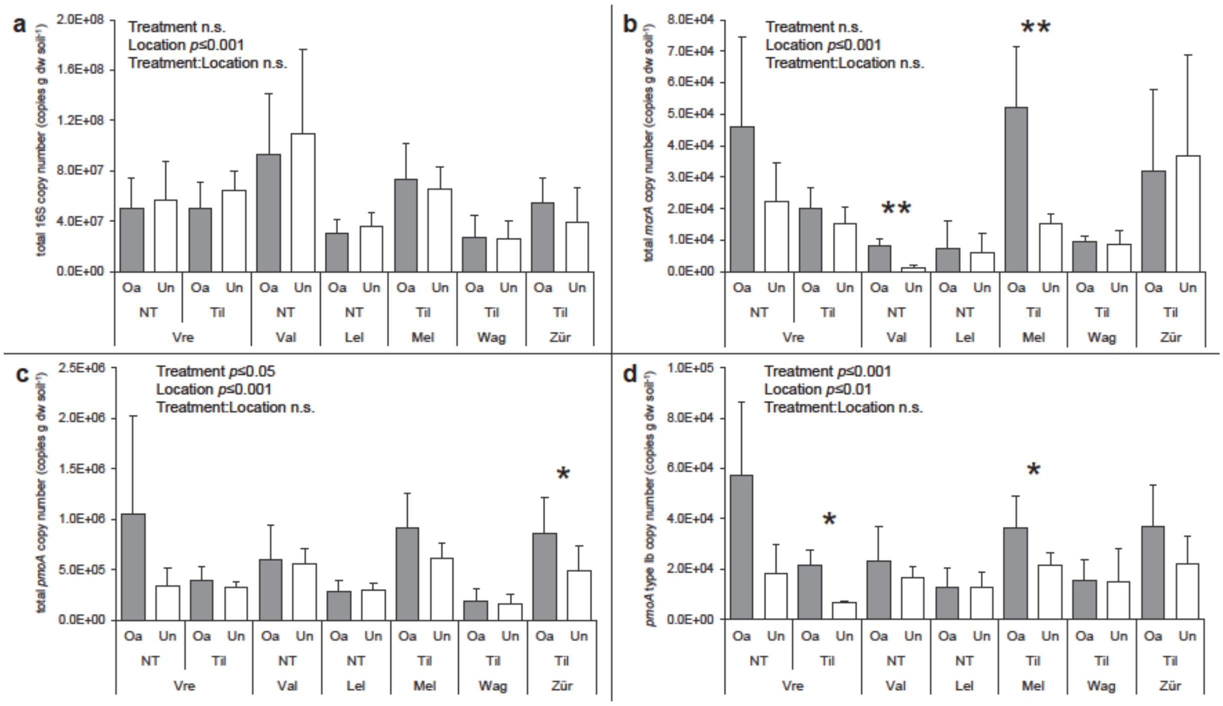

Figure S5. Abundance of 16S (a), *mcrA* (b), total *pmoA* (c), and *pmoA* type Ib (d) genes per gram dry weight soil (mean ± SD; *n*=4-6) of the sub-top layer of agricultural soils (7.5-15 cm depth) of visited field sites (Vre – Vredepeel; Val – Valthermond; Lel – Lelystad; Mel – Melle; Wag – Wageningen; Zür – Zürich), varying in organic treatment (Oa – organic-amended; Un – unamended) and agricultural practice (NT – no-tillage; Til – tillage). Significant differences within a location are indicated with an asterisk (paired t-test; * $p \leq 0.05$; ** $p \leq 0.01$; *** $p \leq 0.001$). Treatment and location effects, and their interaction, are given if significant (one-way ANOVA or Scheirer-Ray-Hare test).

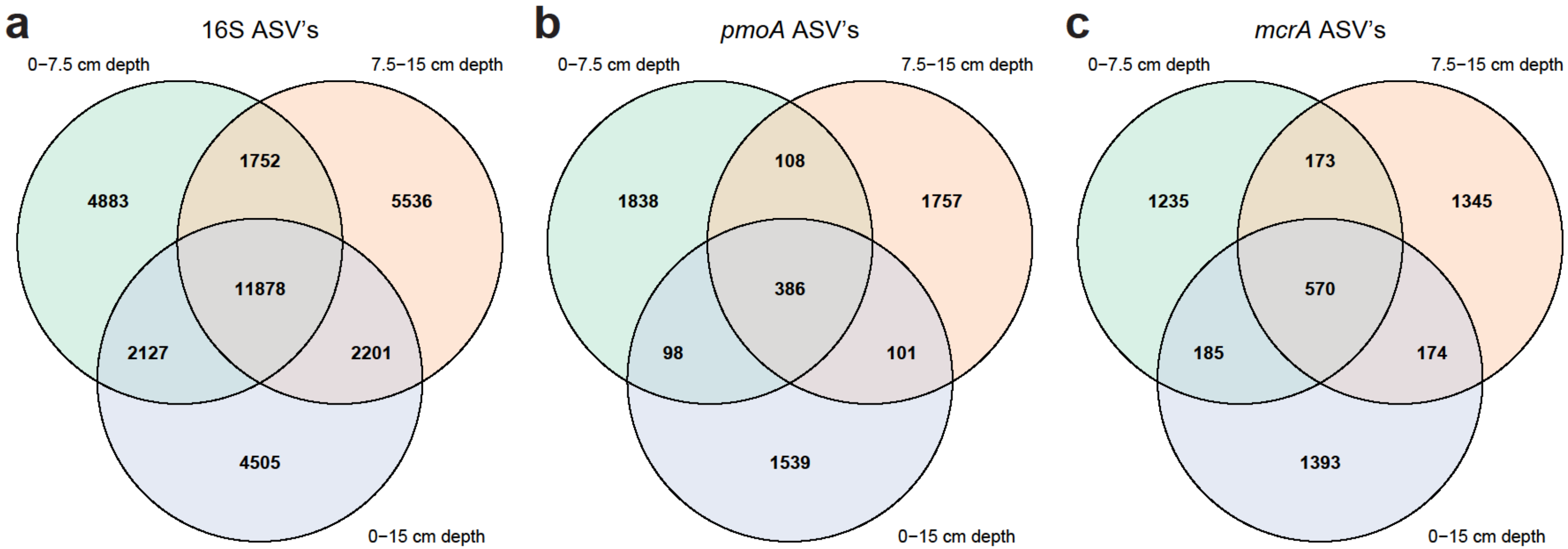

Figure S6. Venn diagram of total identified and classified ASV's of 16S (a), *pmoA* (b), and *mcrA* (c) target genes of amplicon sequencing analysis in agricultural soils (0-15 cm depth), its upper-top layer (0-7.5 cm depth) and its sub-top layer (7.5-15 cm depth).

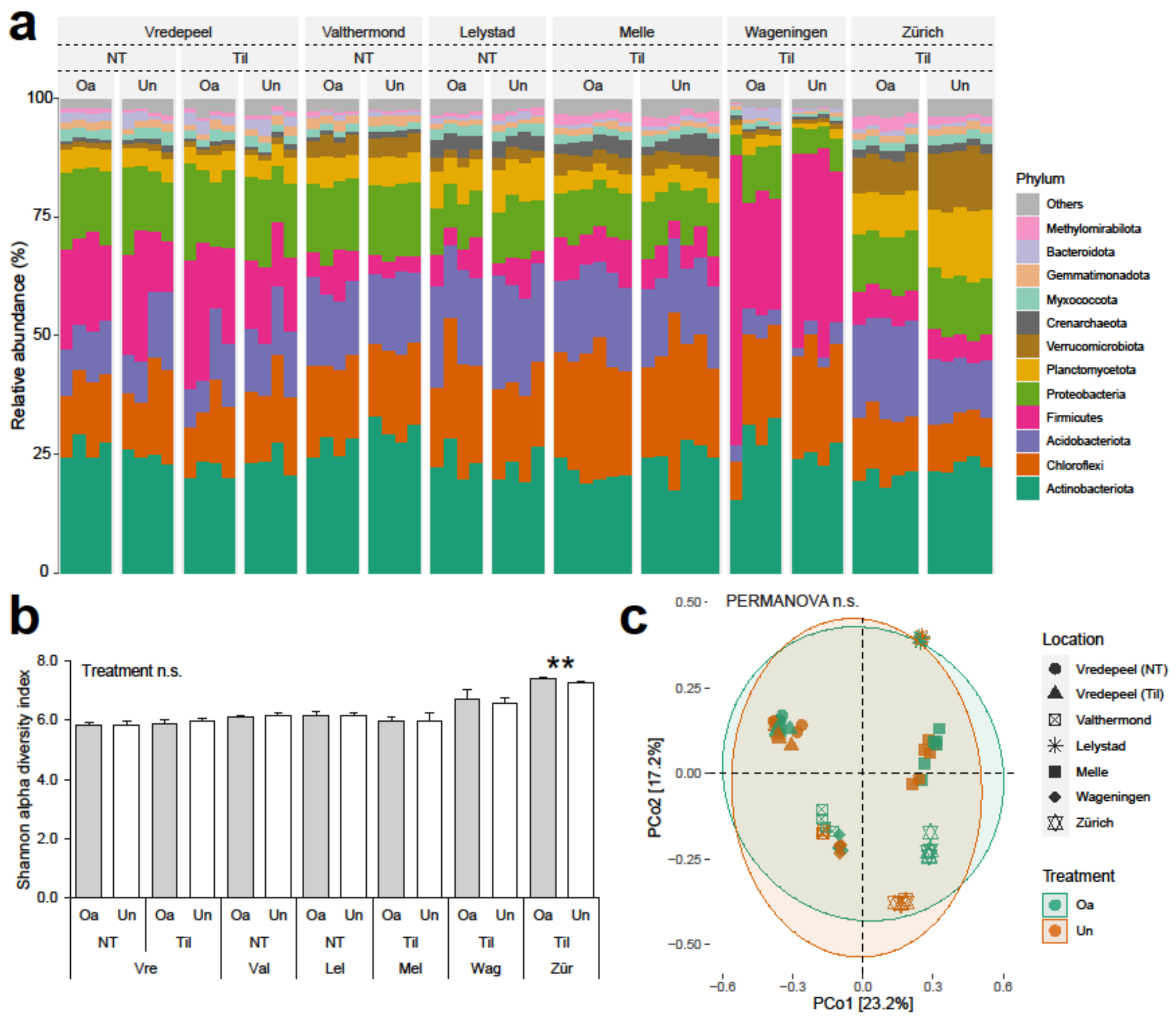

Figure S7. Relative abundance of the total bacterial community composition on species level (a), Shannon alpha diversity (b), and PCoA beta diversity (c) based on bacterial 16S rRNA ASV's of agricultural soils (0-15 cm depth) of visited field sites (Vre – Vredepeel; Val – Valthermond; Lel – Lelystad; Mel – Melle; Wag – Wageningen; Zür – Zürich), varying in organic treatment (Oa – organic-amended; Un – unamended) and agricultural practice (NT – no-tillage; Til – tillage). Significant differences on the alpha diversity within a location are indicated with an asterisk (paired t-test; * $p \leq 0.05$; ** $p \leq 0.01$; *** $p \leq 0.001$), and overall treatment effects on the alpha and beta diversity are given (one-way ANOVA and PERMANOVA, respectively).

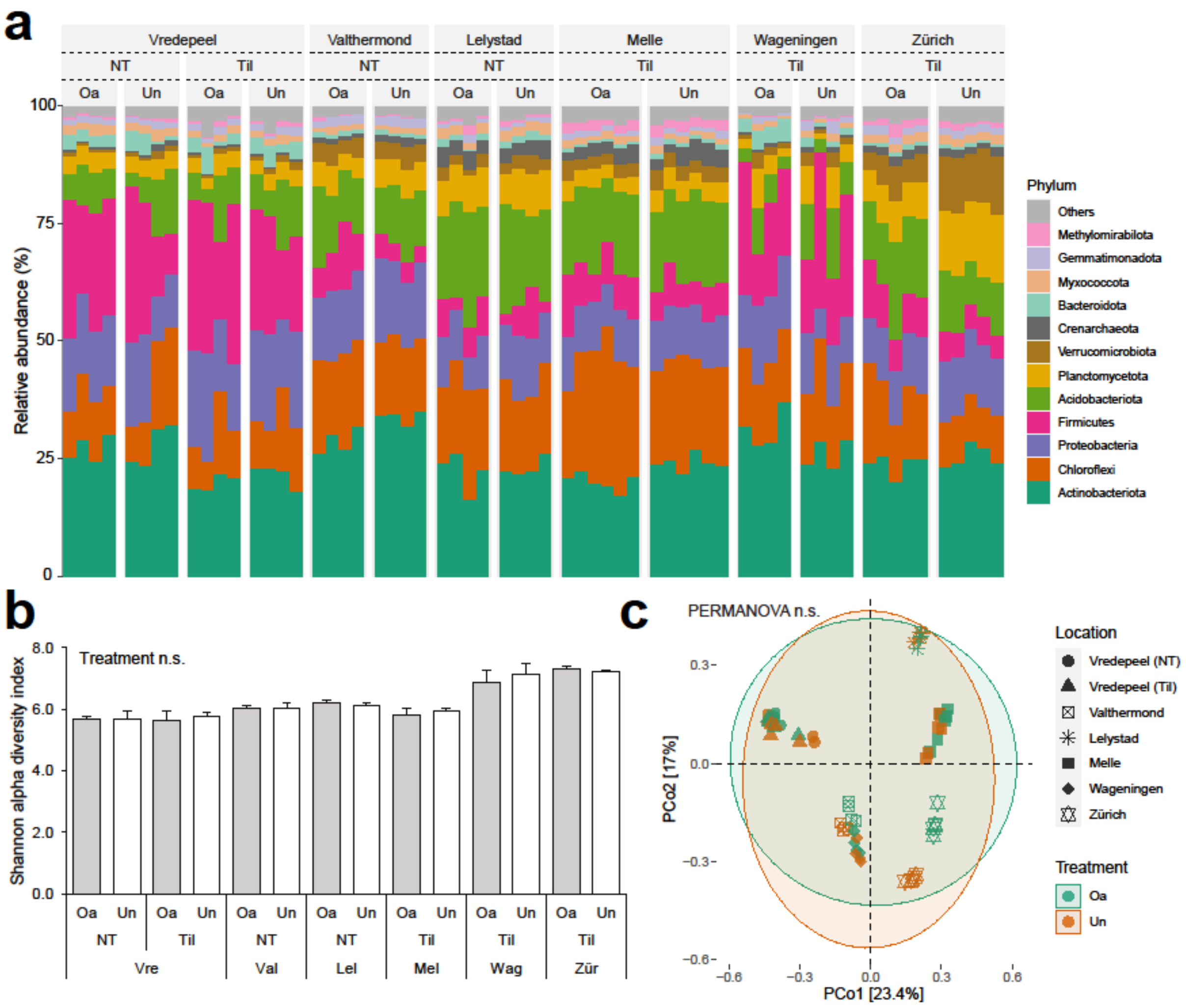

Figure S8. Relative abundance of the total bacterial community composition on species level (a), Shannon alpha diversity (b), and PCoA beta diversity (c) based on bacterial 16S rRNA ASV's of the upper-top layer of agricultural soils (0-7.5 cm depth) of visited field sites (Vre – Vredepeel; Val – Valthermond; Lel – Lelystad; Mel – Melle; Wag – Wageningen; Zür – Zürich), varying in organic treatment (Oa – organic-amended; Un – unamended) and agricultural practice (NT – no-tillage; Til – tillage). Significant differences on the alpha diversity within a location are indicated with an asterisk (paired t-test; * $p \leq 0.05$; ** $p \leq 0.01$; *** $p \leq 0.001$), and overall treatment effects on the alpha and beta diversity are given (one-way ANOVA and PERMANOVA, respectively).

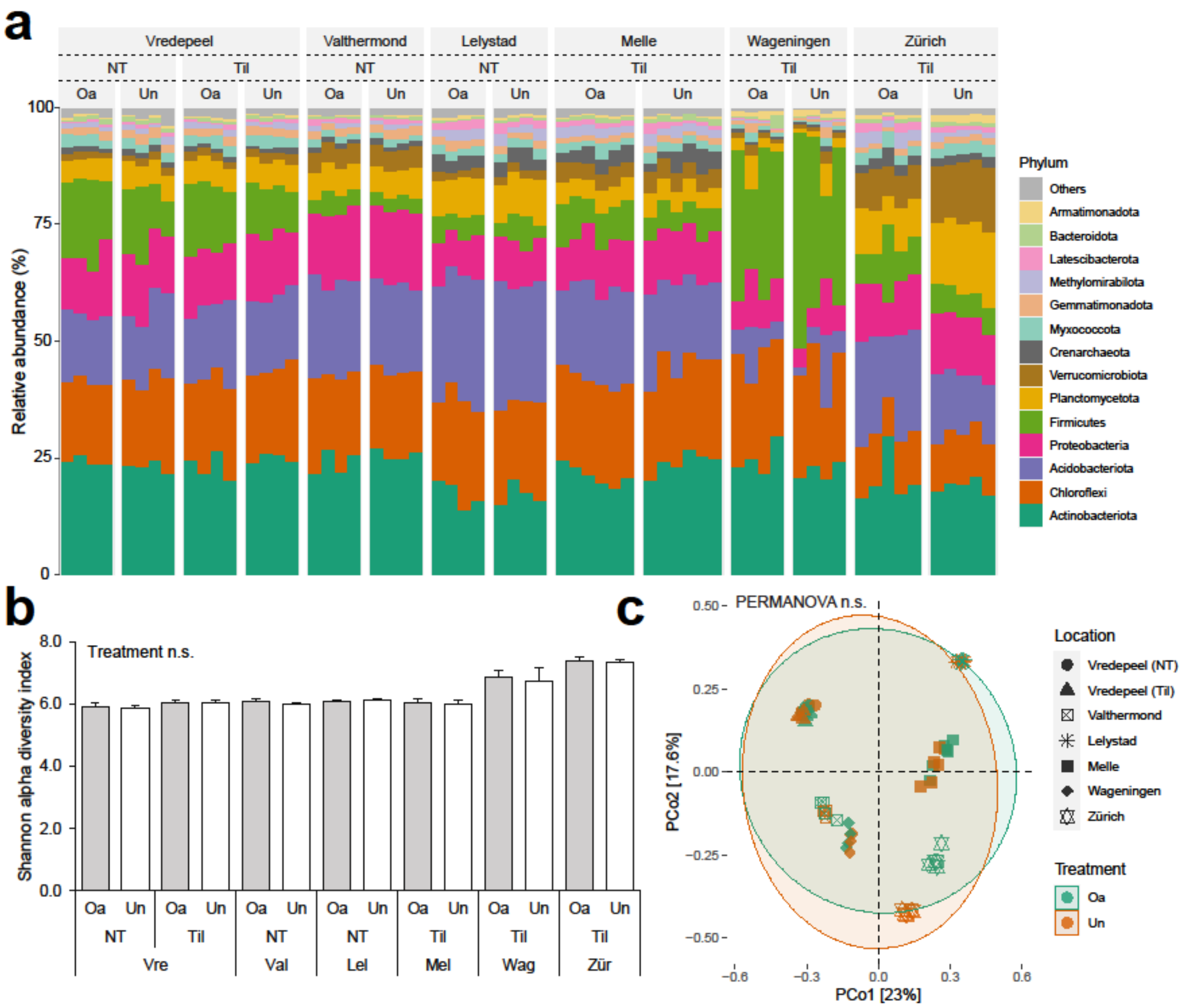

Figure S9. Relative abundance of the total bacterial community composition on species level (a), Shannon alpha diversity (b), and PCoA beta diversity (c) based on bacterial 16S rRNA ASV's of the sub-top layer of agricultural soils (7.5-15 cm depth) of visited field sites (Vre – Vredepeel; Val – Valthermond; Lel – Lelystad; Mel – Melle; Wag – Wageningen; Zür – Zürich), varying in organic treatment (Oa – organic-amended; Un – unamended) and agricultural practice (NT – no-tillage; Til – tillage). Significant differences on the alpha diversity within a location are indicated with an asterisk (paired t-test; * $p \leq 0.05$; ** $p \leq 0.01$; *** $p \leq 0.001$), and overall treatment effects on the alpha and beta diversity are given (one-way ANOVA and PERMANOVA, respectively).

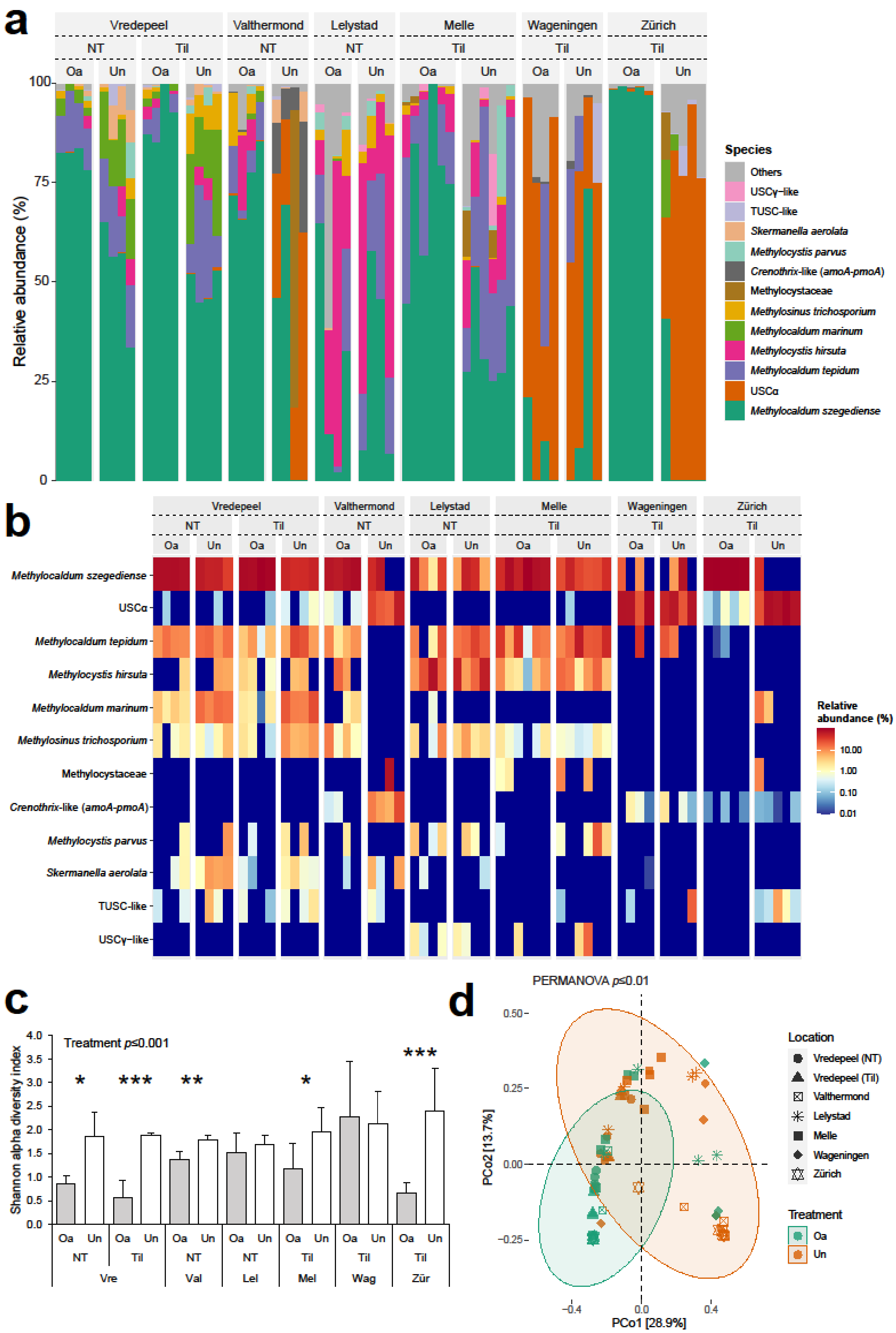
a
Vredepeel
Valthermond
Lelystad
Melle
Wageningen
Zürich
NT
Til
Oa
Un
Relative abundance (%)
100
75
50
25
0
Species
Others
USCγ–like
TUSC-like
Skermanella aerolata
Methylocystis parvus
Crenothrix-like (amoA-pmoA)
Methylocystaceae
Methylosinus trichosporium
Methylocaldum marinum
Methylocystis hirsuta
Methylocaldum tepidum
USCα
Methylocaldum szegediense
b
Relative abundance (%)
10.00
1.00
0.10
0.01
c
Shannon alpha diversity index
Treatment p≤0.001
Vre
Val
Lel
Mel
Wag
Zür
d
PERMANOVA p≤0.01
PCo1 [28.9%]
PCo2 [13.7%]
Location
Vredepeel (NT)
Vredepeel (Til)
Valthermond
Lelystad
Melle
Wageningen
Zürich
Treatment
Oa
Un

Figure S10. Relative abundance of the methanotrophic community composition on species level (a), heatmap of most abundant methanotrophic species (b), Shannon alpha diversity (c), and PCoA beta diversity (d) based on *pmoA* ASV's of the upper-top layer of agricultural soils (0-7.5 cm depth) of visited field sites (Vre – Vredepeel; Val – Valthermond; Lel – Lelystad; Mel – Melle; Wag – Wageningen; Zür – Zürich), varying in organic treatment (Oa – organic-amended; Un – unamended) and agricultural practice (NT – no-tillage; Til – tillage). Significant differences on the alpha diversity within a location are indicated with an asterisk (paired t-test; * $p \leq 0.05$; ** $p \leq 0.01$; *** $p \leq 0.001$), and overall treatment effects on the alpha and beta diversity are given (one-way ANOVA and PERMANOVA, respectively).

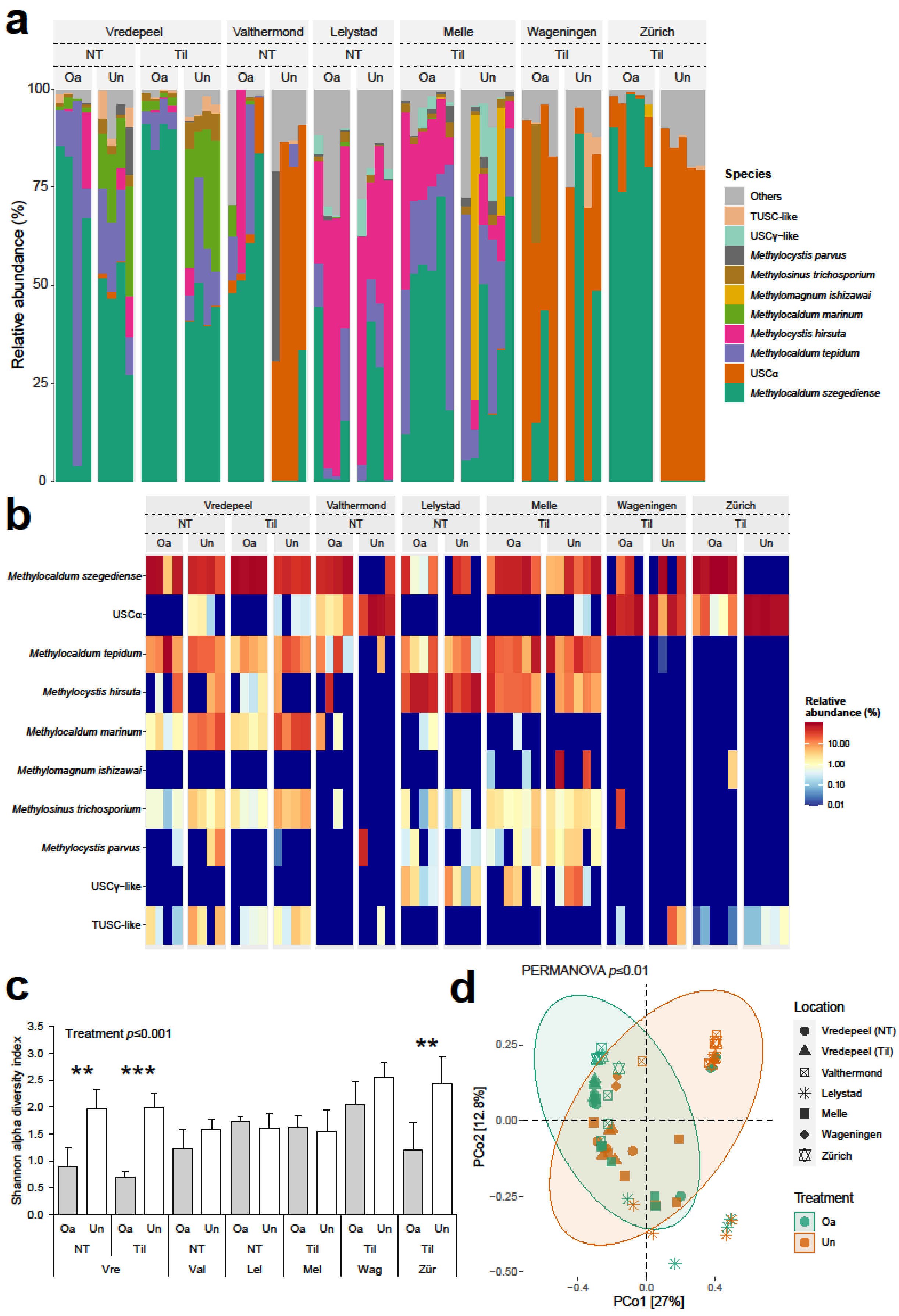
a
Vredepeel
Valthermond
Lelystad
Melle
Wageningen
Zürich
NT
Til
Oa
Un
Relative abundance (%)
100
75
50
25
0
Species
Others
TUSC-like
USCγ-like
Methylocystis parvus
Methylosinus trichosporium
Methylomagnum ishizawai
Methylocaldum marinum
Methylocystis hirsuta
Methylocaldum tepidum
USCα
Methylocaldum szegediense
b
Relative abundance (%)
10.00
1.00
0.10
0.01
c
Treatment p≤0.001
Shannon alpha diversity index
3.5
3.0
2.5
2.0
1.5
1.0
0.5
0.0
**
***
Vre
Val
Lel
Mel
Wag
Zür
d
PERMANOVA p≤0.01
PCo1 [27%]
PCo2 [12.8%]
0.25
0.00
−0.25
−0.50
−0.4
0.0
0.4
Location
Vredepeel (NT)
Vredepeel (Til)
Valthermond
Lelystad
Melle
Wageningen
Zürich
Treatment
Oa
Un

Figure S11. Relative abundance of the methanotrophic community composition on species level (a), heatmap of most abundant methanotrophic species (b), Shannon alpha diversity (c), and PCoA beta diversity (d) based on *pmoA* ASV's of the sub-top layer of agricultural soils (7.5-15 cm depth) of visited field sites (Vre – Vredepeel; Val – Valthermond; Lel – Lelystad; Mel – Melle; Wag – Wageningen; Zür – Zürich), varying in organic treatment (Oa – organic-amended; Un – unamended) and agricultural practice (NT – no-tillage; Til – tillage). Significant differences on the alpha diversity within a location are indicated with an asterisk (paired t-test; * $p \leq 0.05$; ** $p \leq 0.01$; *** $p \leq 0.001$), and overall treatment effects on the alpha and beta diversity are given (one-way ANOVA and PERMANOVA, respectively).

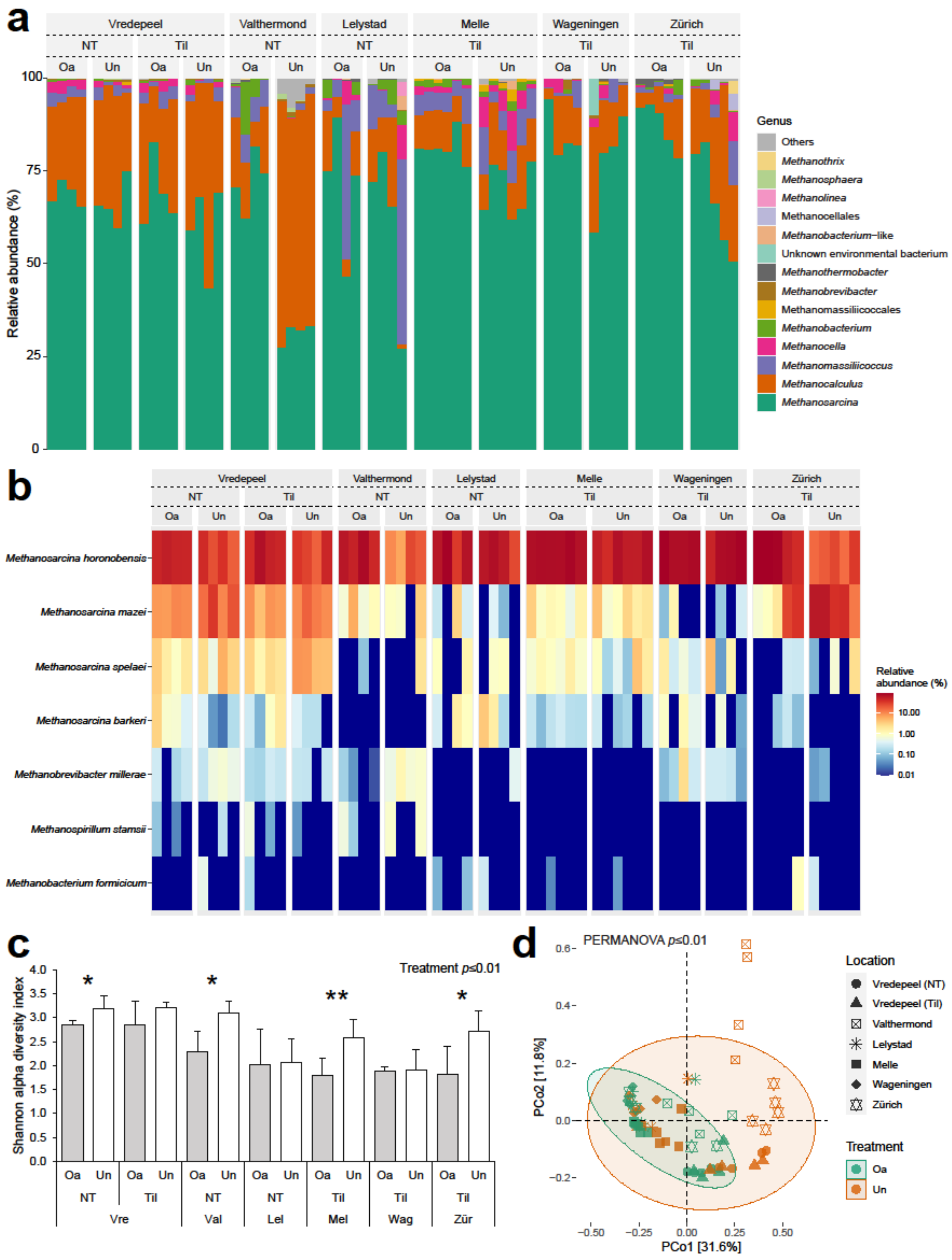

Figure S12. Relative abundance of the methanogenic community composition on genus level (a), heatmap of most abundant methanogenic species (b), Shannon alpha diversity (c), and PCoA beta diversity (d) based on *mcrA* ASV's of the upper-top layer

of agricultural soils (0-7.5 cm depth) of visited field sites (Vre – Vredepeel; Val – Valthermond; Lel – Lelystad; Mel – Melle; Wag – Wageningen; Zür – Zürich), varying in organic treatment (Oa – organic-amended; Un – unamended) and agricultural practice (NT – no-tillage; Til – tillage). Significant differences on the alpha diversity within a location are indicated with an asterisk (paired t-test; * $p \leq 0.05$; ** $p \leq 0.01$; *** $p \leq 0.001$), and overall treatment effects on the alpha and beta diversity are given (one-way ANOVA and PERMANOVA, respectively).

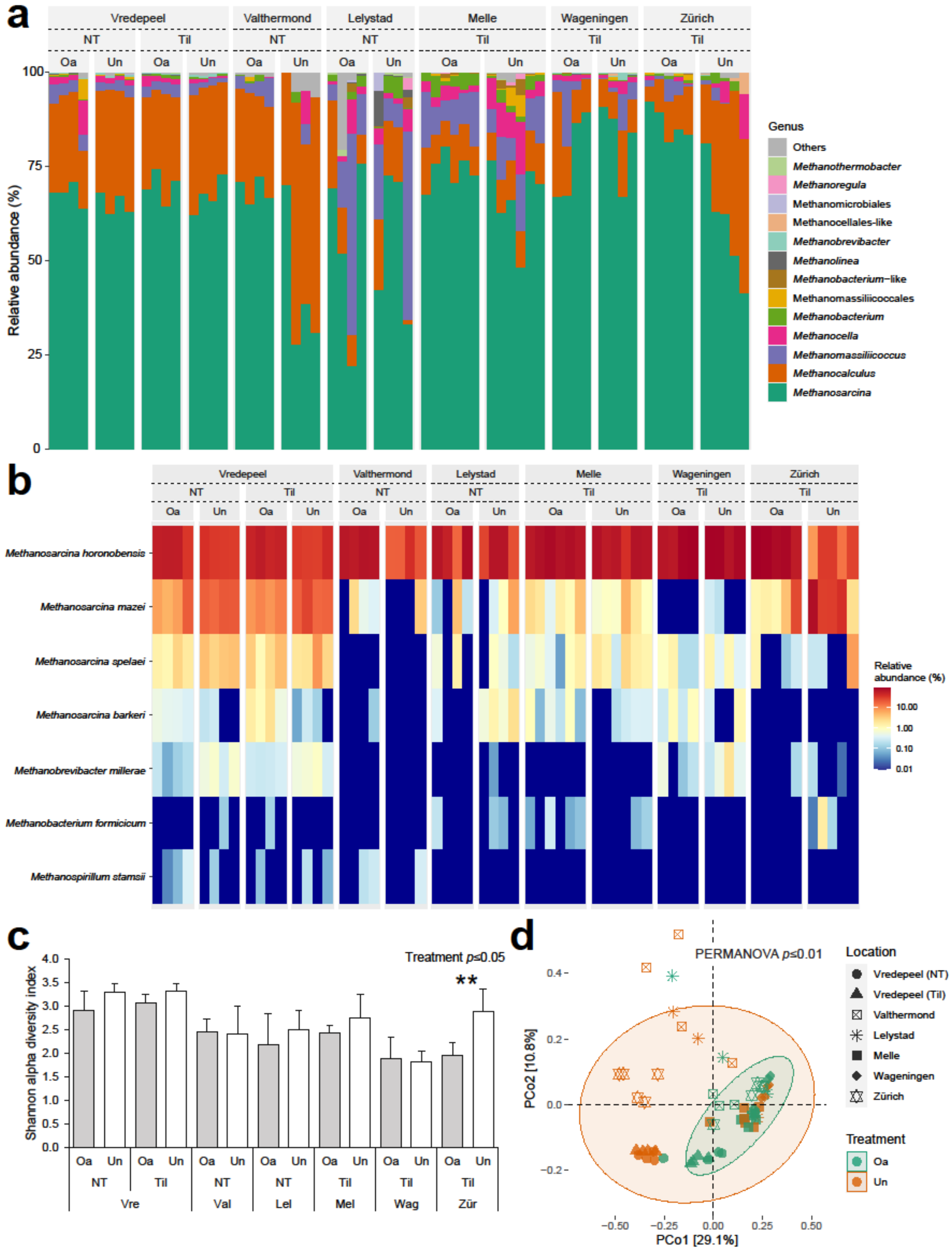

Figure S13. Relative abundance of the methanogenic community composition on genus level (a), heatmap of most abundant methanogenic species (b), Shannon alpha diversity (c), and PCoA beta diversity (d) based on *mcrA* ASV's of the sub-top layer of

agricultural soils (7.5-15 cm depth) of visited field sites (Vre – Vredepeel; Val – Valthermond; Lel – Lelystad; Mel – Melle; Wag – Wageningen; Zür – Zürich), varying in organic treatment (Oa – organic-amended; Un – unamended) and agricultural practice (NT – no-tillage; Til – tillage). Significant differences on the alpha diversity within a location are indicated with an asterisk (paired t-test; * $p \leq 0.05$; ** $p \leq 0.01$; *** $p \leq 0.001$), and overall treatment effects on the alpha and beta diversity are given (one-way ANOVA and PERMANOVA, respectively).

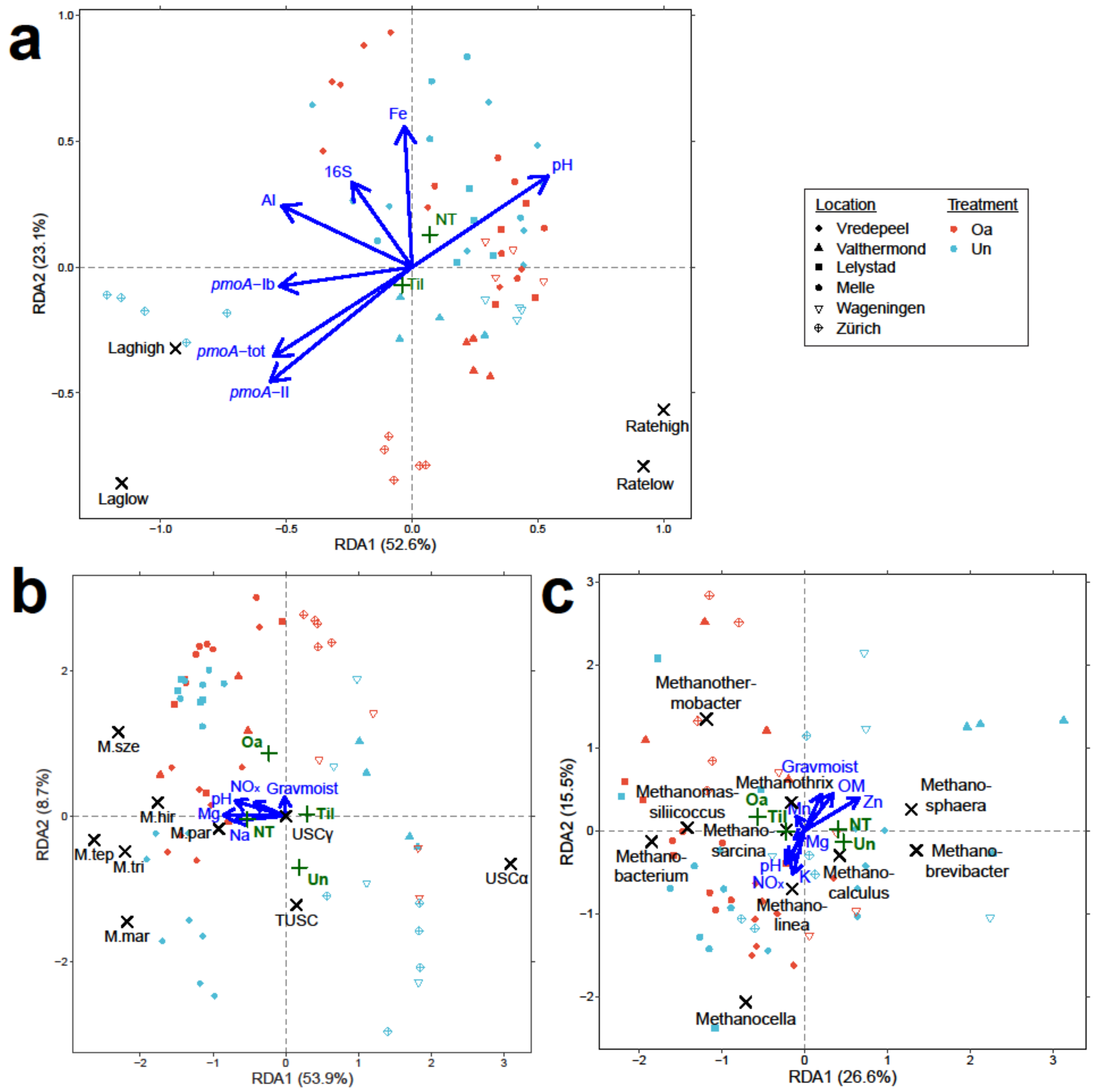

Figure S14. RDA on variables explaining the variability observed in the methane uptake potential (a), the methanotrophic community (b) and the methanogenic community (c) in the upper-top layer of agricultural soils (0-7.5 cm depth), obtained using the environmental variables gravimetric moisture content (Gravmoist), organic matter content (OM), pH, and the $(NO_2^-+NO_3^-)$-N-, Al-, Fe-, K-, Mn-, Mg-, Na-, and Zn-content (respectively NOx, Al, Fe, K, Mn, Mg, Na, Zn), the gene copy numbers of 16S, *pmoA* total (pmoA-tot), *pmoA* type Ib (*pmoA*-Ib), and *pmoA* type II (*pmoA*-II), and the agricultural management variables organic treatment (Oa for organic-amended, Un for

unamended) and agricultural practice (NT for no-tillage, Til for tillage). The methane uptake potential is based on the methane uptake rates and lag phases in incubations with ~10 ppm_v CH_4 (Ratelow and Laglow, respectively) and ~10,000 ppm_v CH_4 (Ratehigh and Laghigh, respectively), the methanotrophic community is based on the species abundance of *Methylocaldum szegediense* (M.sze), Upland Soil Cluster α (USCα), *Methylocaldum tepidum* (M.tep), *Methylocystis hirsuta* (M.hir), *Methylocaldum marinum* (M.mar), *Methylosinus trichosporium* (M.tri), *Methylocystis parvus* (M.par), Tropical Upland Soil Cluster (TUSC), and Upland Soil Cluster γ (USCγ), and the methanogenic community is based on the abundance of the methanogenic genera.

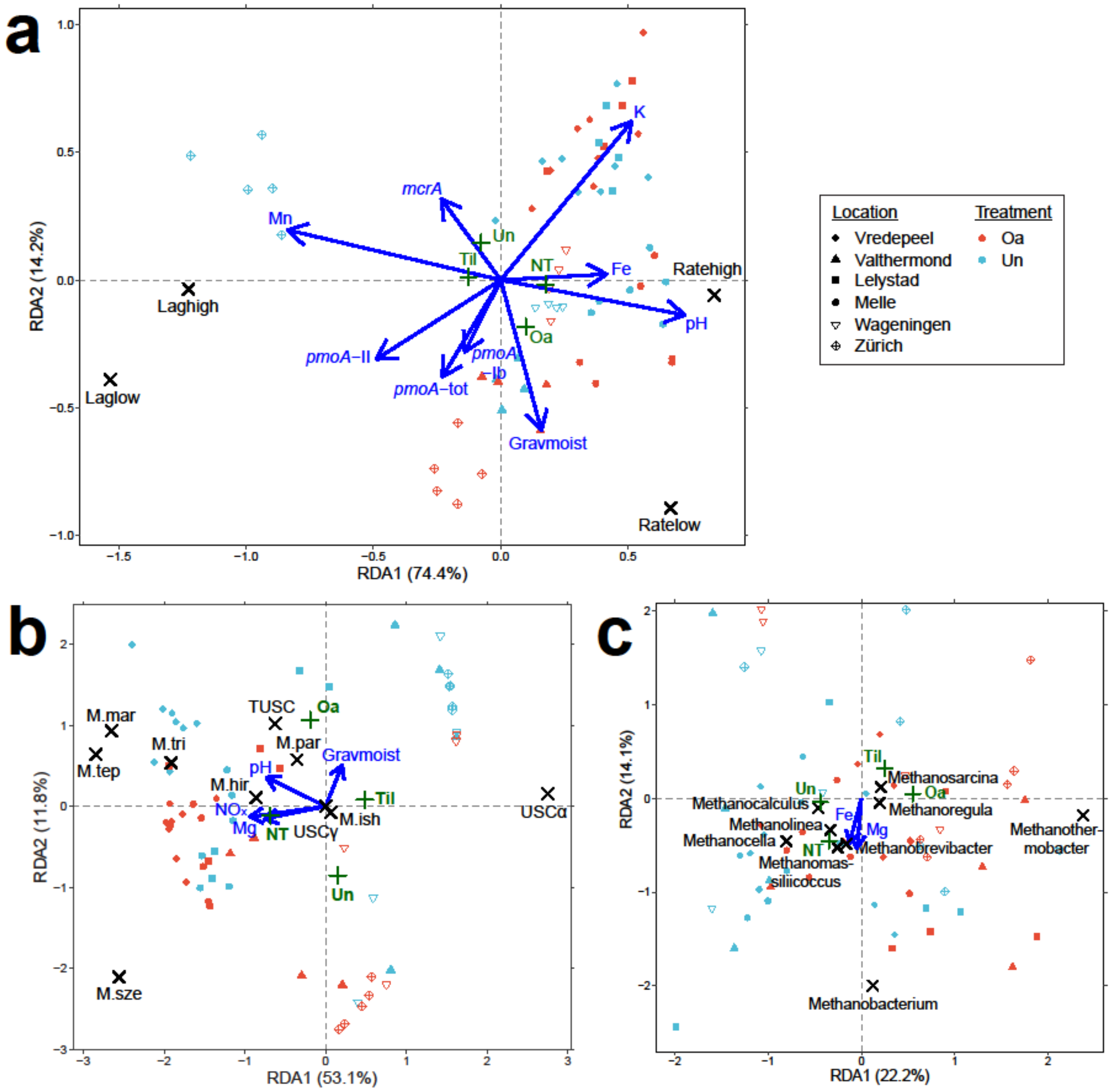

Figure S15. RDA on variables explaining the variability observed in the methane uptake potential (a), the methanotrophic community (b) and the methanogenic community (c) in the sub-top layer of agricultural soils (7.5-15 cm depth), obtained using the environmental variables gravimetric moisture content (Gravmoist), pH, and the (NO_2^-+NO_3^-)-N-, Fe-, K-, Mn-, Mg-content (respectively NOx, Fe, K, Mn, Mg), the gene copy numbers of *pmoA* total (pmoA-tot), *pmoA* type Ib (*pmoA*-Ib), *pmoA* type II (*pmoA*-II) and *mcrA*, and the agricultural management variables organic treatment (Oa for organic-amended, Un for unamended) and agricultural practice (NT for no-tillage,

Til for tillage). The methane uptake potential is based on the methane uptake rates and lag phases in incubations with ~10 ppm_v CH_4 (Ratelow and Laglow, respectively) and ~10,000 ppm_v CH_4 (Ratehigh and Laghigh, respectively), the methanotrophic community is based on the species abundance of *Methylocaldum szegediense* (M.sze), Upland Soil Cluster α (USCα), *Methylocaldum tepidum* (M.tep), *Methylocystis hirsuta* (M.hir), *Methylocaldum marinum* (M.mar), *Methylomagnum ishizawai* (M.ish), *Methylosinus trichosporium* (M.tri), *Methylocystis parvus* (M.par), Upland Soil Cluster γ (USCγ), and Tropical Upland Soil Cluster (TUSC), and the methanogenic community is based on the abundance of the methanogenic genera.

References

1. van den Bergh SG, Chardon I, Leite MFA, Korthals GW, Mayer J, Cougnon M, et al. Soil aggregate stability governs field greenhouse gas fluxes in agricultural soils. *Soil Biol Biochem* 2024; 191: 109354.

2. Bieganowski A, Ryżak M, Sochan A, Barna G, Hernádi H, Beczek M, et al. Laser Diffractometry in the Measurements of Soil and Sediment Particle Size Distribution. *Advances in Agronomy* 2018; 151: 215–279.

3. Faé GS, Montes F, Bazilevskaya E, Añó RM, Kemanian AR. Making Soil Particle Size Analysis by Laser Diffraction Compatible with Standard Soil Texture Determination Methods. *Soil Science Society of America Journal* 2019; 83: 1244–1252.

4. de Boer GBJ, de Weerd C, Thoenes D, Goossens HWJ. Laser Diffraction Spectrometry: Fraunhofer Diffraction Versus Mie Scattering. *Particle & Particle Systems Characterization* 1987; 4: 14–19.

5. Makó A, Tóth G, Weynants M, Rajkai K, Hermann T, Tóth B. Pedotransfer functions for converting laser diffraction particle-size data to conventional values. *Eur J Soil Sci* 2017; 68: 769–782.

6. Polakowski C, Makó A, Sochan A, Ryżak M, Zaleski T, Beczek M, et al. Recommendations for soil sample preparation, pretreatment, and data conversion for texture classification in laser diffraction particle size analysis. *Geoderma* 2023; 430: 1–13.

7. Ryżak M, Bieganowski A. Methodological aspects of determining soil particle-size distribution using the laser diffraction method. *Journal of Plant Nutrition and Soil Science* 2011; 174: 624–633.

8. Soil Science Division Staff. Soil survey manual, USDA Handbook 18. *Soil Conservation Service Volume Handbook 18* . 2017. U.S. Department of Agriculture, Washington, D.C,.

9. R Core Team. R: A Language and Environment for Statistical Computing. 2020. R Foundation for Statistical Computing, Vienna, Austria.

10. Oksanen J, Simpson GL, Blanchet FG, Kindt R, Legendre P, Minchin PR, et al. Package ‘vegan’. *Community Ecology Package* 2022; version 2.6-2: 1–295.

11. Liu C, Cui Y, Li X, Yao M. Microeco: An R package for data mining in microbial community ecology. *FEMS Microbiol Ecol* 2021; 97: fiaa255.

12. Baveye PC, Otten W, Kravchenko A, Balseiro-Romero M, Beckers É, Chalhoub M, et al. Emergent properties of microbial activity in heterogeneous soil microenvironments: Different research approaches are slowly converging, yet major challenges remain. *Front Microbiol* 2018; 9.

13. Kasmerchak CS, Mason JA, Liang M. Laser diffraction analysis of aggregate stability and disintegration in forest and grassland soils of northern Minnesota, USA. *Geoderma* 2019; 338: 430–444.

14. Gyawali AJ, Stewart RD. An Improved Method for Quantifying Soil Aggregate Stability. *Soil Science Society of America Journal* 2019; 83: 27–36.

15. Svensson DN, Messing I, Barron J. An investigation in laser diffraction soil particle size distribution analysis to obtain compatible results with sieve and pipette method. *Soil Tillage Res* 2022; 223: 105450.
